# Mucosal administration of a live attenuated recombinant COVID-19 vaccine protects nonhuman primates from SARS-CoV-2

**DOI:** 10.1038/s41541-022-00509-6

**Published:** 2022-07-29

**Authors:** Mariana F. Tioni, Robert Jordan, Angie Silva Pena, Aditya Garg, Danlu Wu, Shannon I. Phan, Christopher M. Weiss, Xing Cheng, Jack Greenhouse, Tatyana Orekov, Daniel Valentin, Swagata Kar, Laurent Pessaint, Hanne Andersen, Christopher C. Stobart, Melissa H. Bloodworth, R. Stokes Peebles, Yang Liu, Xuping Xie, Pei-Yong Shi, Martin L. Moore, Roderick S. Tang

**Affiliations:** 1 Meissa Vaccines Inc, Redwood City, CA USA; 2 BIOQUAL Inc, Rockville, MD USA; 3 Department of Biological Sciences, Butler University, Indianapolis, IN USA; 4 Division of Allergy, Pulmonary and Critical Care Medicine, Department of Medicine, Vanderbilt University School of Medicine, Nashville, TN USA; 5 Department of Pathology, Microbiology, and Immunology, Vanderbilt University School of Medicine, Nashville, TN USA; 6 Department of Biochemistry and Molecular Biology, University of Texas Medical Branch, Galveston, TX USA; 7 Present Address: Bill & Melinda Gates Foundation, Seattle, WA USA

**Keywords:** Live attenuated vaccines, SARS-CoV-2

## Abstract

Severe acute respiratory syndrome coronavirus 2 (SARS-CoV-2) is the causative agent of the COVID-19 global pandemic. SARS-CoV-2 is an enveloped RNA virus that relies on its trimeric surface glycoprotein spike for entry into host cells. Here we describe the COVID-19 vaccine candidate MV-014-212, a live, attenuated, recombinant human respiratory syncytial virus expressing a chimeric SARS-CoV-2 spike as the only viral envelope protein. MV-014-212 was attenuated and immunogenic in African green monkeys (AGMs). One mucosal administration of MV-014-212 in AGMs protected against SARS-CoV-2 challenge, reducing by more than 200-fold the peak shedding of SARS-CoV-2 in the nose. MV-014-212 elicited mucosal immunoglobulin A in the nose and neutralizing antibodies in serum that exhibited cross-neutralization against virus variants of concern Alpha, Beta, and Delta. Intranasally delivered, live attenuated vaccines such as MV-014-212 entail low-cost manufacturing suitable for global deployment. MV-014-212 is currently in Phase 1 clinical trials as an intranasal COVID-19 vaccine.

## Introduction

COronaVIrus Disease 2019 (COVID-19) is the latest virus pandemic to afflict humanity. The disease began its spread in the late months of 2019^1,2^, and by March 11, 2020, 118,000 people across 114 countries were infected, at which time it was declared a pandemic by the World Health Organization (WHO)^3^. COVID-19 is a respiratory disease often leading to pneumonia, caused by the highly transmissible severe acute respiratory syndrome coronavirus 2 (SARS-CoV-2)^4^. The overall mortality rate is ~2% and the disease is especially severe in the elderly and in patients with serious underlying medical conditions, such as heart or lung disease and diabetes. As of May 8, 2022, there were 515,192,979 confirmed cases of SARS-CoV-2 infection with a total of 6,254,140 deaths worldwide (WHO dashboard, https://covid19.who.int/).

SARS-CoV-2 is an enveloped RNA virus that relies on its surface glycoprotein spike for entry into host cells^5,6^. The spike protein is a type I fusion protein that forms a trimer that protrudes on the viral membrane, giving the virus its characteristic crown-like appearance under electron microscopy^7,8^. The angiotensin-converting enzyme 2 (ACE2) has been identified as a cellular receptor for SARS-CoV-2 spike^5,9^. Disrupting the interaction of ACE2 and the receptor binding domain (RBD) of spike is at the core of vaccine design and therapeutics. Currently, nine COVID-19 vaccines are validated for use by the WHO^10^. Six of these vaccines are based on the SARS-CoV-2 spike protein, and their high level of efficacy has validated spike as a protective antigen.

All the vaccines validated by the WHO are delivered intramuscularly, and none is live attenuated. Live attenuated vaccines (LAVs) are often administered by the same route of entry as the pathogen they target, and replicate in the host, mimicking natural infection without causing disease. For respiratory viruses, intranasal LAVs generate mucosal immunity at the site of infection, blocking the pathogen at the earliest phases of infection thus helping control systemic spread^11^. In the case of Influenza infection, LAV induces better mucosal immunoglobulin A (IgA) and cell-mediated immunity relative to other vaccine types, eliciting a longer-lasting and broader immune response that more closely resembles natural immunity^12^. In SARS-CoV-2 infection, the early antibody response is dominated by IgA and mucosal IgA is highly neutralizing^13^, underscoring the importance of developing an intranasal vaccine capable of eliciting mucosal immunity. Furthermore, comparison of intramuscular vs. intranasal vaccines in mice showed that serum IgA was only induced following intranasal vaccination against SARS-CoV^14^ and only intranasal vaccination provided protection from SARS-CoV-2 in both upper and lower respiratory tracts^15^. A recent comparison of the protection afforded by the COVID-19 vaccine ChAdOx1 nCoV-19/AZD1222 in hamsters, showed that after challenge with SARS-CoV-2 the animals vaccinated via the intranasal route shed less virus in the nose compared to the animals vaccinated intramuscularly^16^. According to the WHO vaccine tracker, there are currently 140 vaccines in clinical trials, only four of which are intranasal replicating vaccines^17^.

Here we describe the rational design, generation, and preclinical evaluation of a novel COVID-19 vaccine candidate. MV-014-212 is a live attenuated recombinant vaccine strain derived from the human respiratory syncytial virus (RSV) vaccine candidate OE4^18^. In MV-014-212, the RSV attachment (G) and fusion (F) surface glycoproteins were replaced with a chimeric SARS-CoV-2 spike harboring the cytoplasmic tail of the RSV F protein. MV-014-212 was attenuated and immunogenic in nonhuman primates (NHPs), producing both systemic and mucosal immunity after one mucosal administration. A single mucosal administration of MV-014-212 in African green monkeys (AGMs) elicited neutralizing antibodies against the homologous virus and cross-neutralizing activity against the variants of concern, Alpha (B.1.1.7), Beta (B.1.351), and Delta (B.1.617.2). Studies in mice indicated that MV-014-212 vaccination generated a cellular immune response biased towards a type 1 T helper (Th1) cell response. MV-014-212 is currently being evaluated in Phase 1 clinical trials as an intranasal COVID-19 vaccine.

## Results

### Design and generation of MV-014-212

**Fig. 1:**
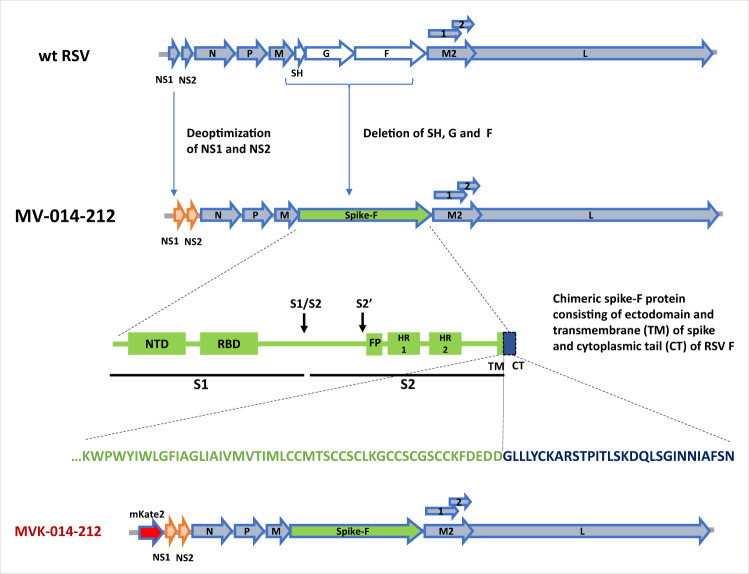
Design of MV-014-212. In MV-014-212, the *NS1* and *NS2* genes are codon deoptimized and the respiratory syncytial virus (RSV) *SH*, *G*, and *F* genes are deleted and replaced by a gene encoding a chimeric Spike protein. The amino acid sequence at the junction is shown. The transmembrane domain of spike is represented in green, and the cytoplasmic tail of F is depicted in blue. The reporter virus MVK-014-212, encoding the fluorescent protein mKate2 in the first gene position, is schematically shown at the bottom of the panel. FP, fusion peptide; HR1 and 2, heptad repeats 1 and 2; NTD, N-terminal domain; RBD, receptor binding domain; S1, subunit S1; S2, subunit S2; S1/S2 and S2’, protease cleavage sites; wt, wild type.

MV-014-212 is a novel, live attenuated, recombinant vaccine against SARS-CoV-2, based on the backbone of the human respiratory syncytial virus (RSV) (Fig. 1). The G and F proteins of RSV were replaced by a chimeric protein consisting of the ectodomain and transmembrane (TM) domains of SARS-CoV-2 spike (USA-WA1/2020) and the cytoplasmic tail of RSV F (strain line 19). The rational design of this chimera was based on previous observations of the need for the homologous TM of spike in coronavirus infectious particle production^19–21^ and the reported importance of the cytoplasmic tail of RSV F in the production of RSV progeny^22^. The sequence of amino acids at the junction between spike and F proteins is shown in Fig. 1. Notably, the chimeric spike/RSV F protein retains functionality as MV-014-212 relies on it for attachment and fusion with the host cell. Moreover, since this is the only surface protein of MV-014-212, interference of vaccine take due to preexisting antibodies against RSV is not a concern and cellular immunity against wild-type RSV is not expected to inhibit the replication of MV-014-212 in nasal tissues because natural and experimental RSV reinfection are well documented in literature^23,24^. Various chimeric spike constructs that differed in the SARS-CoV-2 and RSV junction position were assessed for growth in Vero cells (Supplementary Fig. 1). Of note, a construct with the entire native SARS-CoV-2 spike was evaluated (MV-014-300, Supplementary Fig. 1). While this construct could be rescued, it did not propagate productively in cell culture, demonstrating that the cytoplasmic tail of the F protein contributes to viral growth of the chimeric virus. Of the constructs expressing different chimeric spike/RSV F fusion proteins, MV-014-212 was selected for further evaluation based on the ease of rescue and its ability to grow to acceptable titers for preclinical and clinical studies.

The RSV backbone used to generate MV-014-212 was attenuated for replication in primary airway cells by codon deoptimization of the genes encoding the proteins NS1 and NS2 that suppress host innate immunity^25^. In addition, the short hydrophobic glycoprotein SH was deleted (Fig. 1) to further attenuate the virus in vivo and increase transcription of downstream genes^18^.

To facilitate research and assay development, a reporter virus derived from MV-014-212 was constructed by inserting the gene encoding the fluorescent mKate2 protein^26,27^ upstream of the *NS1* gene (MVK-014-212, Fig. 1).

**Fig. 2:**
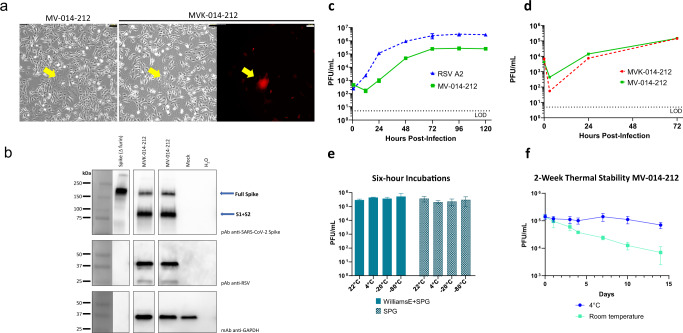
In vitro characterization of MV-014-212. **a** Syncytia formed by MV-014-212 and MVK-014-212. Micrographs were taken at a total amplification of 100× under phase contrast or using tetramethylrhodamine filter. Yellow arrows point to syncytia. Scale bars are 100 µm. **b** Western blot showing full-length purified SARS-CoV-2 spike protein lacking the furin cleavage site (lane 1), MVK-014-212 (lane 2), MV-014-212 (lane 3), mock-infected Vero cell lysate (lane 4), blank (lane 5). The molecular weight markers correspond to the migration of the BIO-RAD Precision Plus Protein Dual Color Standards. The blue arrows indicate the expected size of the full-length spike and the cleaved protein (S1 + S2). The blots were derived from the same experiment and processed in parallel. **c** Multicycle replication kinetics of MV-014-212 compared to respiratory syncytial virus (RSV) A2 in serum-free Vero cells. Cells were infected at an MOI of 0.01 and incubated at 32 °C. Cells and supernatants were collected at 0, 12, 24, 48, 72, 96, and 120 h post infection. Titers of the samples were determined by plaque assay in Vero cells. Data points represent the means of two replicate wells and error bars represent the standard deviation. **d** Multicycle replication kinetics of MV-014-212 compared with MVK-014-212 in serum-free Vero cells. Cells were infected at an MOI of 0.01 and incubated at 32 °C. Cells and supernatants were collected at 0, 3, 24, and 72 h post infection. Titers of the samples were determined by plaque assay in Vero cells. Data points represent the means of three replicate wells and error bars represent the standard deviation. **e** Short-term thermal stability assay. Virus stocks of MV-014-212 prepared in Williams E medium supplemented with sucrose phosphate glutamate (SPG) buffer or prepared in SPG alone were incubated for 6 h at −80 °C, −20 °C, 4 °C, and room temperature and the titer determined by plaque assay. The bars show the mean of 2 technical replicates of 3 different samples, and the error bars denote SD. **f** Two-week thermal stability assay. Virus stocks of MV-014-212 in Williams E medium supplemented with SPG buffer were incubated for 14 days at 4 °C and room temperature (18 °C to 22 °C ± 2 °C) and the titer determined by plaque assay. The bars show the mean of 2 technical replicates of 3 different samples, and the error bars denote SD.

The recombinant virus constructs were electroporated into Vero cells and infectious virus was rescued and propagated for further characterization, as described previously^27^. In MV-014-212, cytopathic effect (CPE) is observed as the formation of polynucleated bodies or syncytia and eventual cell detachment (Fig. 2a). The electroporated cells were expanded until the CPE was extensive and the virus stock was harvested as a total cell lysate. The titers obtained for MV-014-212 and MVK-014-212 were comparable and within the range 1–5 × 10^5^ PFU/mL.

### In vitro characterization of MV-014-212

The SARS-CoV-2 spike protein contains a cleavage site between the S1 and S2 domains that is processed by furin-like proteases^28^ (Fig. 1). As for other coronaviruses, the S1 and S2 subunits of SARS-CoV-2 spike are believed to remain noncovalently bound in the prefusion conformation after cleavage^29,30^. To determine if the chimeric spike protein encoded by MV-014-212 is expressed and proteolytically processed, virus stocks prepared from lysates of infected Vero cells were analyzed on western blots and probed with polyclonal antiserum against SARS-CoV-2 spike protein. Both MV-014-212 and MVK-014-212 viruses express the full length and cleaved forms of the chimeric spike protein (Fig. 2b and Supplementary Fig. 2), consistent with partial cleavage at the S1–S2 junction, with the expected apparent sizes^31,32^ (Supplementary Fig. 2).

Multicycle growth kinetics of MV-014-212 was compared to wild-type (*wt*) recombinant RSV A2 in Vero cells (Fig. 2c). MV-014-212 exhibited delayed growth kinetics relative to RSV A2, showing an initial lag phase of ~12 h. Both viruses reached their peak titers at 72 hpi and the titers remained constant until 120 hpi. The peak titer for MV-014-212 was approximately one order of magnitude lower than that of RSV A2. To determine if the insertion of the *mKate2* gene affected replication kinetics of MVK-014-212, Vero cells were infected with MV-014-212 or MVK-014-212. The growth kinetics of MVK-014-212 was similar to that of MV-014-212, reaching comparable peak titers by 72 hpi (Fig. 2d). These data are consistent with a report that insertion of *mKate2* in the first gene position did not significantly attenuate RSV A2-line19F in vitro^27^.

To evaluate the short-term thermal stability of MV-014-212, aliquots of the viral stock were incubated at different temperatures for a period of 6 h and titered by plaque assay. Two stocks of MV-014-212 prepared in different excipients were compared in this study (Fig. 2e). The results demonstrate that MV-014-212 is stable for at least 6 h in either excipient at –80 °C and room temperature. The stability of MV-014-212 at room temperature (18 °C–22 °C ± 2 °C) and 4 °C was further studied for 15 days. The results shown in Fig. 2f show that MV-014-212 retains its infectivity for 10 days at 4 °C with approximate 0.3 log reduction at day 15.

The genetic stability of MV-014-212 was examined by serial passaging in Vero cells. Sub-confluent Vero cells were infected in triplicate with an aliquot of MV-014-212 and passaged for 10 consecutive passages. Viral RNA was isolated from passages 0 and 10 and amplified by reverse transcription polymerase chain reaction (RT-PCR). The sequence of the entire coding regions of the viral genome was determined by Sanger sequencing. The results showed that for all three lineages there were no variations detected at passage 10 relative to the starting stock (passage 0). Thus, the vaccine candidate was highly stable genetically in vitro.

An antigenic characterization of MV-014-212 confirmed that the spike encoded by the vaccine virus is recognized by monoclonal neutralizing antibodies against SARS-CoV-2 spike (Supplementary Fig. 3) and bound human ACE2 (Supplementary Fig. 3).

### MV-014-212 replication is attenuated in African green monkeys and confers protection against *wt* SARS-CoV-2 challenge

Given the chimeric nature of MV-014-212, the optimal animal model to study its replication and immunogenicity should be permissive to both SARS-CoV-2 and RSV infection. Because there are no known animal models that are fully permissive to both SARS-CoV-2 and RSV, African green monkeys (AGMs) were selected as the best semi-permissive model. AGMs are semi-permissive for replication of both *wt* SARS-CoV-2^33–35^ and RSV^36^, and therefore are an appropriate NHP model for studying the attenuation and protective immunogenicity of MV-014-212.

**Fig. 3:**
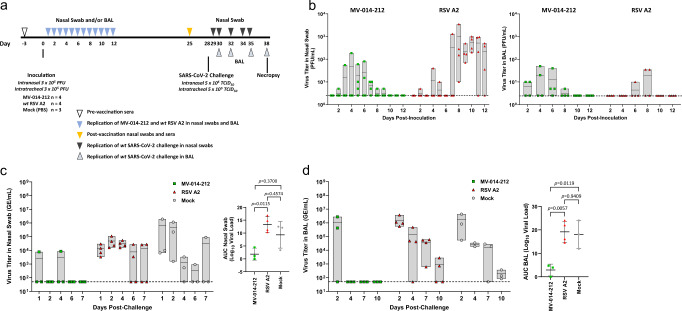
MV-014-212 attenuation in African green monkeys (AGMs) and protection against SARS-CoV-2 challenge. **a** AGM study design. Nasal swabs (NS) were obtained on days 1 through 12 after vaccination. Bronchoalveolar lavages (BALs) were collected on days 2, 4, 6, 8, 10, and 12. Viral shedding of the vaccine or control virus in NS and BAL samples was determined by plaque assay using fresh samples. On day 28 post-inoculation, AGMs were challenged with wild type (*wt*) SARS-CoV-2. Nasal swabs were obtained on days 1, 2, 4, 6, and 7 post challenge. BAL was obtained on days 2, 4, 7, and 10 post challenge. **b** Attenuation of MV-014-212 in the upper and lower respiratory tract of AGM. Viral titer in NS (left) or BAL (right) from AGMs following inoculation with MV-014-212 or *wt* respiratory syncytial virus (RSV) A2 were measured by plaque assay on Vero cells. Each data point is the mean of 2 technical replicates. The box represents the range of the data and the horizontal line is the mean value for each time point. The dotted line represents the limit of detection (50 PFU/mL). *N* = 4 AGMs for each group. For shedding kinetics of individual animals see Supplementary Fig. 7. Protection of MV-014-212-vaccinated AGMs against *wt* SARS-CoV-2 challenge in nasal swabs (**c**) and BAL (**d**). SARS-CoV-2 sgRNA levels were measured by RT-qPCR and expressed as genome equivalents (GE)/mL. The horizontal line in the box is the mean of all measurements at each time point. The dashed line represents the limit of detection of 50 GE/mL. Each data point corresponds to one animal (*N* = 3 AGMs for MV-014-212 and mock groups and *N* = 4 AGMs for RSV A2 group). For shedding kinetics of individual animals see Supplementary Fig. 9. Statistical analysis was one-way ANOVA with Tukey’s multiple comparisons. *P*-values are shown. Bars are mean and error bars denote SD.

The AGM study design is depicted in Fig. 3a. On day 0, AGMs were inoculated via the intranasal (IN) and intratracheal (IT) routes with 1.0 mL of 3 × 10^5^ PFU/mL MV-014-212 or *wt* RSV A2 at each site for a total dose of 6 × 10^5^ PFU per animal. Due to the semi-permissive nature of the AGM model, IT inoculation was necessary to promote replication of the vaccine and the SARS-CoV-2 challenge virus in the lungs. Animals in the mock group were similarly inoculated with phosphate-buffered saline (PBS). Body weight and temperature were recorded for each animal daily (Supplementary Figs. 5, 6). Nasal swabs (NS) and bronchoalveolar lavage (BAL) samples were collected through day 12 after immunization. Viral shedding in NS and BAL samples was determined by plaque assay using fresh samples that were not frozen at the study site. The results showed that the level of infectious virus in animals inoculated with MV-014-212 and duration of shedding in nasal secretions were lower than in animals inoculated with RSV A2 (Fig. 3b and Supplementary Fig. 7). The mean peak titer for RSV A2 was ~20-fold higher than that observed for animals inoculated with MV-014-212. These results show that MV-014-212 is attenuated in the upper respiratory tract of AGMs, compared to RSV A2.

Low to undetectable virus titers were also observed in the lower respiratory tract of animals inoculated with MV-014-212 or RSV A2 over the course of 12 days. Both viruses replicated at low levels, but peak levels occurred earlier for MV-014-212. In this study, RSV A2 showed 100- to 1000-fold lower peak titers in the lower respiratory tract of AGMs compared with *wt* RSV A2 titers reported in literature^37–40^, confounding the ability to assess attenuation of MV-014-212 in the lungs. Subsequently, lower titers were also observed in the lungs of cotton rats for the recombinant A2 used in this study (rA2 from Meissa Vaccines Inc., Supplementary Fig. 8), relative to biologically derived RSV strains, suggesting that the RSV A2 used in this AGM study was attenuated in the lungs of AGMs.

Nasal and BAL samples from day 6 post vaccination were used to extract RNA for sequence analyses of the spike gene of MV-014-212. Using Sanger sequencing, no variations in the spike gene were detected compared with the reference sequence for MV-014-212.

Consistent with the overall low levels of MV-014-212 replication in the respiratory tract of AGMs, no adverse events that were considered treatment-related were observed following inoculation with the vaccine. On day 16 post vaccination, one monkey inoculated with MV-014-212 died unexpectedly. Death occurred 4 days after the last NS and BAL surgical sample collection. The results of a necropsy performed by an ACLAM-certified veterinarian indicated that a definitive cause of death could not be ascertained based on macroscopic or microscopic postmortem evaluations; however, there was no evidence that suggested the death was vaccine-related. Moreover, the deceased animal had the lowest MV-014-212 titer in NS samples compared to the other animals in this treatment group, with only one swab containing virus that was above the detection limit of the plaque assay (50 PFU/mL) on day 5, and no detectable infectious virus in BAL at any of the time points evaluated (Supplementary Fig. 7, AGM C7675).

On day 28, AGMs were challenged with 10^6^ median tissue culture infectious dose (TCID_50_) of *wt* SARS-CoV-2. Body weight and temperature were recorded for each animal daily after challenge (Supplementary Figs. 5, 6). NS and BAL samples were collected for 7 and 10 days after challenge respectively. Shedding of *wt* SARS-CoV-2 was measured by quantitative RT-qPCR of the *E* gene subgenomic SARS-CoV-2 RNA (sgRNA) (Fig. 3c, d and Supplementary Fig. 9). Total viral shedding after challenge was captured as the area under the curve (AUC) for each individual animal across all measured time points and compared between treatment groups.

MV-014-212-vaccinated monkeys had low or undetectable levels of *wt* SARS-CoV-2 sgRNA in NS samples, in contrast to animals inoculated with *wt* RSV A2 or PBS (mock), which had higher levels of SARS-CoV-2 sgRNA. While the level of SARS-CoV-2 sgRNA was undetectable in animals vaccinated with MV-014-212 at most time points, AGM C6672 had detectable SARS-CoV-2 sgRNA at day 1 and AGM C8959 had a similar titer at day 4 post challenge (Supplementary Fig. 9). Mean peak titers of SARS-CoV-2 in NS of animals in the control RSV and mock groups were 20- and 250-fold higher than for animals vaccinated with MV-014-212, respectively. In both RSV- and mock-infected animals, shedding of *wt* SARS-CoV-2 sgRNA did not increase or decreased steadily in nasal secretions from days 4 to 7. The mean AUC for shedding in the nose of the MV-014-212 group was cumulatively 7-log lower than the mock group and 11-log lower than the RSV control group.

Vaccination with MV-014-212 resulted in faster clearance of SARS-CoV-2 in lungs compared to RSV A2 or mock group (Fig. 3d and Supplementary Fig. 9). The peak titer of SARS-CoV-2 in BAL samples occurred at day 2 and was similar in all three treatment groups. Lung titers were undetectable in MV-014-212-vaccinated animals on days 4 through 10 whereas SARS-CoV-2 was readily measured in animals inoculated with RSV A2 or PBS. The mean AUC for shedding in the BAL of the MV-014-212 group was cumulatively more than 15-log lower than both the mock and RSV control groups.

Shedding of infectious SARS-CoV-2 in BAL and NS after challenge was quantified by a TCID_50_ assay (Supplementary Fig. 10). The results showed that AGMs vaccinated with MV-014-212 had on average between 100- and 1000-fold less infectious SARS-CoV-2 in BAL, relative to RSV A2 and PBS controls, at peak shedding days. The kinetics of clearance of the MV-014-212 group was also faster, based on the sgRNA qPCR assay. In NS, the average peak shedding of the MV-014-212 group was more than 1000-fold lower than that of the RSV A2 group (Supplementary Fig. 10). In the NS of the mock-vaccinated group, however, one of the animals did not have detectable shedding of infectious virus at any of the time points (Supplementary Fig. 10). As a result, the overall average peak shedding was only 30-fold higher in the mock group than that seen in the MV-014-212-vaccinated group.

Taken together, these data show that a single mucosal administration of MV-014-212 protected AGMs from *wt* SARS-CoV-2 challenge.

### Antibody responses to MV-014-212 vaccination in AGMs

**Fig. 4:**
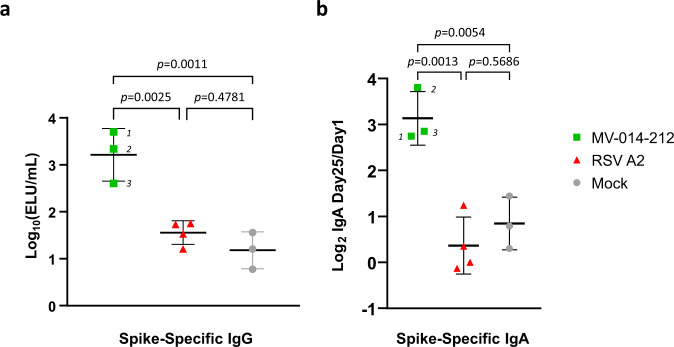
Spike-specific antibody responses in MV-014-212-inoculated African green monkeys (AGMs). **a** Spike-specific serum immunoglobulin (Ig) G antibodies were measured by ELISA using serum collected on day 25 from AGMs inoculated with MV-014-212, wild-type respiratory syncytial virus (RSV) A2, or phosphate-buffered saline (mock). The titer was expressed as ELISA units (ELU/mL) calculated by comparison against a standard curve generated with a pool of human COVID-19 convalescent serum (Std/012020, Nexelis). **b** IgA antibodies specific to SARS-CoV-2 spike protein were measured by ELISA using nasal swabs collected on day 25 post-inoculation. The Log_2_ of the ratio of the values obtained at day 25 over day 1 are shown. The calculated ELU/mL concentration was obtained from a standard curve generated using total purified human IgA by a capture ELISA. For (**a**) and (**b**), *N* = 3 AGMs for the MV-014-212 and mock groups and *N* = 4 AGMs for the RSV A2 group. Statistical analysis was one-way ANOVA using Tukey’s multiple comparisons test. *p-*values are shown. Vaccine responses for individual AGMs are indicated as (1) O9720, (2) C8959, and (3) C6672 in panel (**a**) and (**b**). Bars are mean and error bars denote SD.

**Fig. 5:**
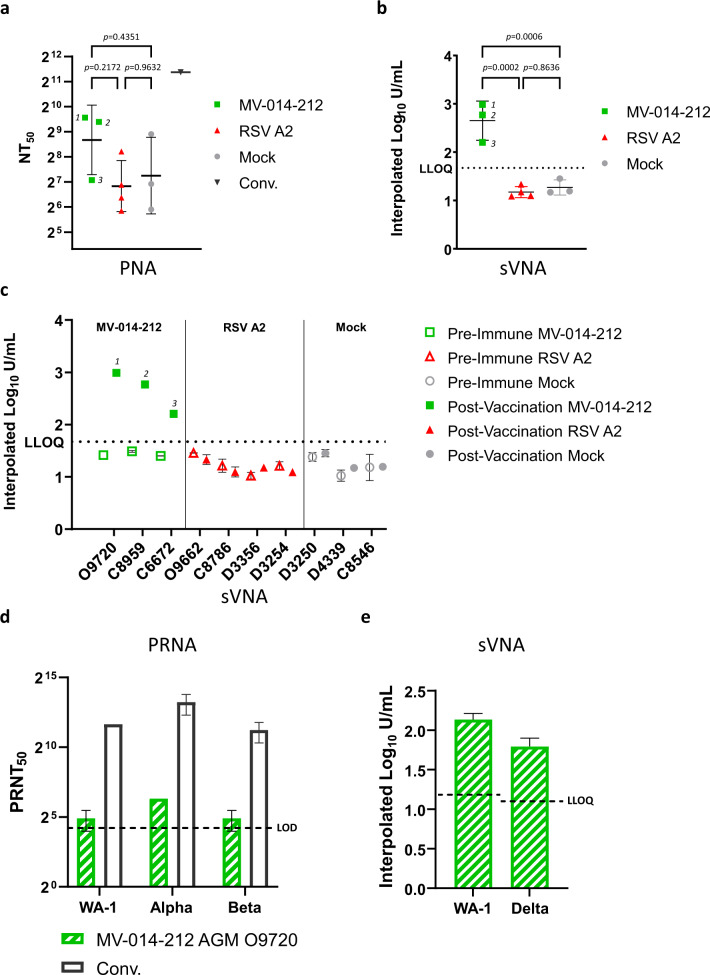
Neutralization assays with African green monkey (AGM) sera. Neutralization assays using day 25 sera from MV-014-212-vaccinated AGMs (1) 09720, (2) C8959, and (3) C6672. **a** Pseudovirus neutralization assay (PNA) showing NT_50_ values and a convalescent sera control at 1859 IU/mL. The data shown is the average of two technical replicates, bars are geometric mean and error bars indicate geometric standard deviation. **b** Surrogate virus neutralization assay (sVNA) results using the USA-WA1/2020 strain RBD. The data shown is the average of two technical replicates, bars are mean and error bars indicate standard deviation. **c** Pre-immune and post-vaccination surrogate virus neutralization titers of individual AGMs. Each data point represents the mean of two technical replicates, except for post-vaccination D3356, D3254, D4339, and C8546. For these animals, only one technical replicate was tested due to insufficient sample volume. Error bars denote SD. **d** Plaque reduction neutralization assay (PRNA). Serum from the AGM O9720, showing the highest titers in (**a**) and (**b**) was used in a 50% plaque-reduction neutralization test performed in a biosafety level 3 facility. The neutralization titers of this AGM against wild-type SARS-CoV-2 or two variants of concern (Beta/B.1.351 and Alpha/B.1.1.7) are shown. The convalescent serum used in this assay is the same as in (**a**). The limit of detection is 20 and is indicated as a dashed line. **e** Surrogate virus neutralization results using the Delta/B.1.617.2 strain RBD or the USA-WA1/2020 (WA-1) RBD on serum from the AGM O9720. For (**d**) and (**e**), the data shown is the average of two technical replicates and bars indicate standard deviation. Statistical analysis was one-way ANOVA using Tukey’s multiple comparisons test. *p*-values are shown. LOD: limit of detection. LLOQ: lower limit of quantification.

SARS-CoV-2 spike-specific serum immunoglobulin G (IgG) and nasal IgA were measured by ELISA in sera and NS, respectively, from AGMs immunized with MV-014-212, RSV A2, or PBS on day 25 post immunization. All animals were seronegative for RSV A2 and SARS-CoV-2 at the start of the study (Fig. 5c and Supplementary Fig. 4). AGMs inoculated with MV-014-212 produced higher levels of SARS-CoV-2 spike-specific IgG in serum compared to AGMs inoculated with RSV A2 or PBS, which had levels of spike-specific IgG that were close to the limit of detection (Fig. 4a).

Spike-specific IgA was also detected in the nasal swabs of monkeys inoculated with MV-014-212 (Supplementary Fig. 11). There was more than an 8-fold increase in nasal spike-specific IgA in the MV-014-212-vaccinated animals 25 days after vaccination (Fig. 4b). In contrast, RSV A2 or mock-vaccinated animals did not show a comparable change in spike-specific IgA.

These results showed that mucosal inoculation of MV-014-212 induced both nasal and systemic antibody responses to the functional SARS-CoV-2 spike.

To determine if vaccination with MV-014-212 elicited neutralizing antibodies in AGMs, different assays were used: (a) luciferase-based pseudovirus neutralization assay (PNA) with S-pseudotyped vesicular stomatitis virus (VSV), (b, c and e) surrogate virus neutralization assay based on ACE2 binding (sVNA), and (d) plaque reduction neutralization assay with SARS-CoV-2 viruses (PRNA). The results of the PNA and sVNA showed that MV-014-212-vaccinated animals had higher levels of serum neutralizing antibodies than RSV and mock-inoculated groups but failed to achieve statistical significance due to overall high level of noise (Fig. 5a, b). The PNA was used to compare the NT_50_ values of the AGM serum samples with human convalescent serum using a commercial pool of three human convalescent sera of titer 1859 UI/mL (Fig. 5a). The AGM sera had on average 4-fold lower titers than the convalescent control. Last, the serum of AGM O9720 vaccinated with MV-014-212, was used to study cross-reactivity with different variants of concern. Cross-reactivity against the Alpha and Beta variants was studied using a conventional 50% plaque-reduction neutralization assay with SARS-CoV-2 in biosafety level 3 containment^41,42^ (Fig. 5d) and the neutralization of Delta was investigated with a sVNA (Fig. 5e). The results showed that vaccination with MV-014-212 elicits neutralizing activity against the *wt* strain and three variants of concern analyzed. In the PRNA, the serum from the MV-014-212-vaccinated AGM O9720 showed activity against the original USA-WA1/2020, Alpha and Beta strains (the pre-immune serum titers were below the limit of detection for all virus strains tested). The results of the sVNA (Fig. 5e) show detectable titers against the Delta variant. No quantitative comparisons can be drawn from the titers of the different variants. Taken together, the data suggested that MV-014-212 elicited modest serum neutralizing antibody titers in AGMs, and there was cross-neutralization against the variants Alpha, Beta, and Delta.

### MV-014-212 elicits a Th1-biased cellular immune response in hACE2-mice

Mouse models of vaccine-associated enhanced respiratory disease (VAERD) suggest that an imbalance in Th1 and type 2 (Th2) T helper cell immunity with a skewing towards Th2 response contributes to enhanced lung pathology following challenge^43^. To assess the balance of Th1 and Th2 immunity generated after vaccination with MV-014-212, transgenic mice expressing human ACE2 receptor were inoculated with a single dose of MV-014-212 or PBS by the intranasal route. A control group received an intramuscular prime and boost vaccination with SARS-CoV-2 spike protein formulated in alum, which has been shown to skew immunity toward a Th2 response^44^. On day 28, serum was collected to measure spike-specific IgG2a, and IgG1 by ELISA. In addition, spleens were collected and the number of splenocytes expressing interferon-γ (IFNγ) or interleukin (IL)-5 were measured by ELISpot assay. The ratio of IgG2a/IgG1 and the ratio of cells producing IFNγ/IL-5 are indicators of Th1-biased cellular immune response^44,45^.

**Fig. 6:**
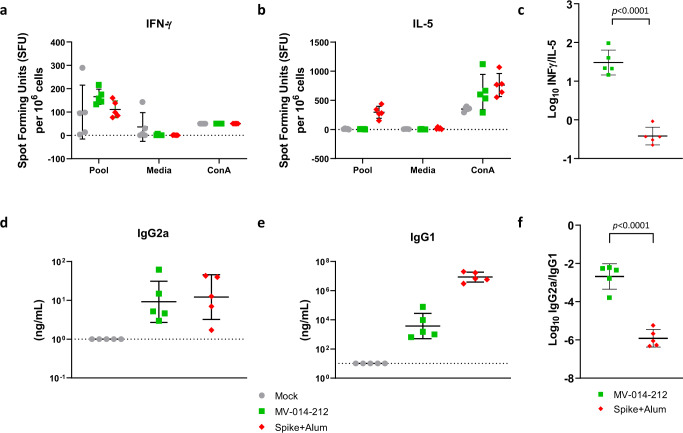
MV-014-212 elicited a type 1 T helper cell-biased immune response in human ACE2 transgenic mice. **a** Interferon (IFN)-γ and **b** interleukin (IL)-5 expressing T cells were quantified by ELISpot. Splenocytes isolated from mice expressing human ACE2 (*N* = 5 mice per group) were collected on day 28 post-inoculation with MV-014-212 or phosphate-buffered saline (PBS) and stimulated with a peptide pool that spanned the SARS-CoV-2 spike protein (pool), media, or the mitogen concanavalin A (Con A). Control mice were vaccinated with purified SARS-CoV-2 spike protein adjuvanted with alum by intramuscular injection at day −20 and day 0. **c** Log of the ratio of IFN-γ to IL-5–expressing cells. Immunoglobulin levels of **d** IgG2a and **e** IgG1 from day 28 serum were determined by Spike-specific ELISA. The concentration of each immunoglobulin isotype was determined from standard curves generated with purified SARS-CoV-2 spike specific monoclonal IgG2a or IgG1 antibodies. **f** Log of the ratio of IgG2a/IgG1. Each data point corresponds to one animal. For (**a**), (**b**), (**c**), and (**f**), the horizontal bar marks the mean and error bars are SD. For (**d**) and (**e**), the horizontal bars are the geometric mean and error bars are geometric SD. Statistical analysis is an unpaired *t* test.

The results showed that MV-014-212 induced spike-reactive splenocytes as measured by ELISpot assay (Fig. 6a–c). Importantly, MV-014-212 induced higher numbers of splenocytes expressing IFNγ relative to IL-5 when cell suspensions were stimulated with a spike peptide pool, suggesting that vaccination with MV-014-212 produced a Th1-biased immune response. The ratio of IFNγ-producing cells to IL-5-producing cells in the MV-014-212 group was more than one order of magnitude higher than in the group vaccinated with alum-adjuvanted spike protein (Fig. 6c). Consistent with the ELISpot data, the ratios of IgG2a/IgG1 detected in serum were higher in the animals vaccinated with MV-014-212 than the control group vaccinated with alum-adjuvanted spike (Fig. 6d–f). These data suggest that intranasal vaccination with live, attenuated, recombinant MV-014-212 induced a Th1-biased antiviral immune response.

## Discussion

MV-014-212 is a recombinant, live, attenuated COVID-19 vaccine designed to be administered intranasally to stimulate mucosal as well as systemic immunity against SARS-CoV-2. MV-014-212 was engineered to express a functional SARS-CoV-2 spike protein in place of the RSV membrane surface proteins F, G, and SH in an attenuated RSV strain expressing codon deoptimized *NS1* and *NS2* genes. Indeed, replication of MV-014-212 was attenuated in the respiratory tract of AGMs following mucosal administrations in the nose and trachea and it elicited SARS-CoV-2 spike-specific mucosal IgA and serum IgG. Furthermore, vaccination with MV-014-212 induced a modest level of serum neutralizing antibodies and, more importantly, a single administration of the Meissa COVID-19 vaccine provided robust protection against SARS-CoV-2 challenge in AGMs.

Based on the levels of viral sgRNA detected after SARS-CoV-2 challenge, the efficacy of MV-014-212 after one dose in nonhuman primates (NHPs) is comparable with that reported for the mRNA vaccines in NHPs. The efficacy and kinetics of clearance in MV-014-212-vaccinated AGMs are comparable to those observed after 2 doses of BNT126b2 or mRNA1273^46,47^ in nasal swabs and BAL of rhesus macaques. Promising preclinical data for two other IN vaccines were recently published^16,48^.

Due to the lack of a fully permissive animal model to both SARS-CoV-2 and RSV, AGMs were selected as a compromise to study the replication and immunogenicity of the chimeric virus MV-014-212 and recapitulate some aspects of disease caused by SARS-CoV-2 infection. However, the use of AGMs as an animal model has practical limitations. First, the availability of AGMs limited the number of animals used and, consequently, diminished the statistical power of this study. While this did not preclude the analysis of the primary immunological endpoints, the small number of AGMs used prevented some of the assays performed in this study from reaching statistical significance. Second, the semi-permissive nature of the animal model for both RSV and SARS-CoV-2 limits the effective potency of an LAV like MV-014-212, which relies on replication to elicit a robust immune response. In a more permissive host like humans, MV-014-212 is expected to replicate to higher titers, which in turn would lead to better immunogenicity than that observed in AGMs.

Despite the limitations addressed above, immunization of AGMs with MV-014-212 resulted in detectable mucosal and systemic antibody responses against spike. The detection of IgA is interesting because an RSV experimental human challenge study showed that low RSV F-specific mucosal IgA was a better predictor for susceptibility to RSV challenge in RSV seropositive adults than serum IgG and neutralizing antibody levels^49^. Furthermore, S RBD-specific dimeric serum IgA was shown to be more potent at neutralizing SARS-CoV-2 than monomeric IgG^50^. By inference, secretory IgA, which exists at mucosal surfaces as dimeric IgA, may act as a potent inhibitor of SARS-CoV-2 at the site of infection. Interestingly, IgA antibodies were shown to dominate early humoral responses in human SARS-CoV-2 infections and IgA plasmablasts with mucosal homing potential peaked during the third week of disease onset^13^, highlighting the role of IgA in natural immunity against SARS-CoV-2. While IgA is an important biomarker of immune response in the nose, its role as a correlate of protection requires further studies.

SARS-CoV-2 neutralizing antibody responses were detected in the serum of vaccinated AGMs by a luciferase-based pseudovirus assay (PNA), a surrogate virus neutralization assay (sVNA), and in a conventional plaque reduction assay (PRNA) with SARS-CoV-2. The PRNA detected a neutralizing response against two variants of concern, Beta (B.1.351) and Alpha (B.1.1.7), as well as the USA-WA1/2020 strain, while the sVNA showed that Spike-specific antibodies elicited by MV-014-212 also neutralized the Delta variant (B.1.617.2). However, the small number of animals in these studies limited the statistical interpretation of the results. Direct comparison of the AGM serum nAb titers to serum nAb titers published for nonreplicating vaccines in rhesus monkeys or to titers in human convalescent sera is confounded by the semi-permissiveness of AGM for MV-014-212 replication. The immunogenicity of MV-014-212 is currently being studied in an ongoing Phase 1 clinical trial.

The intramuscularly administered vaccines currently in use offer high levels of protection, but their ability to prevent asymptomatic transmission is still unclear (reviewed by Tiboni et al.^51^). A recent report from the Centers for Disease Control and Prevention^52^ showed that in breakthrough infections, the viral load in the noses of vaccinated individuals is as high as that of unvaccinated subjects. This finding and the rapid spread of the highly transmissible Delta and Omicron variants underscore the urgent need for a vaccine that has the potential to block infection in the nose and reduces transmission. Vaccines delivered intranasally stand a higher chance of eliciting local mucosal immunity, preventing not only systemic infection but also local replication and shedding. Indeed, intranasal-delivered MV-014-212 resulted in the production of spike-specific IgA in the noses of AGMs. According to the July 30, 2021, WHO report^17^, “The landscape of candidate vaccines in clinical development,” there are nine intranasal COVID-19 vaccines in clinical trials, only two of which are live attenuated viruses, MV-014-212 and COVI-VAC (Codagenix)^53^. Unlike COVI-VAC, MV-014-212 is a nonsegmented negative-strand RNA virus not prone to recombine in nature. RNA recombination is extremely rare for nonsegmented negative-strand RNA viruses outside of experimental co-infections in laboratory settings^54–56^. In addition to the lack of natural recombination, the results presented in this study showed that MV-014-212 was genetically stable. Neither accumulation of mutations nor loss of the furin cleavage site was detected when the virus was serially passaged 10 times in Vero cells. This contrasts with reports of mutations arising in another recombinant live viral COVID-19 vaccine based on the VSV backbone^57^ and *wt* SARS-CoV-2^31^ or pseudotyped SARS-CoV-2^58,59^ propagated in tissue cultures. MV-014-212 virus shed from vaccinated AGMs had no nucleotide changes in the *Spike* gene. Therefore, the chimeric *Spike* gene in MV-014-212 appears to have a stable genotype in vitro and in vivo.

The vaccine profile of MV-014-212 remains unique among the currently approved COVID-19 vaccines and vaccines that are in clinical development. MV-014-212 is administered intranasally, a needle-free route that offers potential advantages for global immunization. The intranasal route is similar to the natural route of infection of SARS-CoV-2 and generates both mucosal and humoral immune responses in AGMs without any adjuvant formulation. Modeling based on yields from production of Phase 1 clinical study material projected a potential dose output of hundreds of millions of doses per annum in a modestly sized facility using high-intensity bioreactor systems. Mucosally delivered live attenuated vaccines such as MV-014-212 entail minimum downstream processing and have an anticipated low production cost. In addition, needle-free delivery reduces supply risks, and the thermal stability profile of MV-014-212 suggests that a formulation that allows for less stringent storage requirements than currently approved vaccines is possible. Overall, MV-014-212 is well-suited for domestic and global deployment as a primary vaccine or as a heterologous booster. MV-014-212 is currently being evaluated as an intranasal vaccine in a Phase 1 clinical trial (NCT04798001).

## Methods

### Cells, viruses, and animals

Vero reference cell bank (RCB)1 (WHO Vero RCB 10-87) cells were grown in minimal essential medium (MEM, Gibco, Thermo-Fisher Scientific) containing 10% fetal bovine serum (FBS, Corning) and 1× Corning Antibiotic/Antimycotic mix consisting of 100 IU/mL penicillin, 100 µg/mL streptomycin, 0.25 µg/mL amphotericin, with 0.085 g/L NaCI. RCB2 cells were derived from RCB1 and adapted to grow in serum-free media. RCB2 cells used in this study were grown in serum-free medium OptiPro (Gibco) supplemented with 4 mM of L-glutamine (Gibco). Both Vero cell lines were cultured at 37 °C, 5% CO_2_, with 95% humidity.

AGMs (*Chlorocebus aethiops*) were obtained from St. Kitts and were of indeterminate age, weighing 3–6 kg. The monkeys were screened and verified to be seronegative for RSV and SARS-CoV-2 by an RSV microneutralization assay and spike SARS-CoV-2 ELISA (BIOQUAL), respectively. Animals also underwent a physical examination by the veterinary staff to confirm appropriate health status prior to study. Each AGM was uniquely identified by a tattoo. One female and three males were assigned to the MV-014-212 and RSV groups. Two males and one female were assigned to the mock group. Cage-side observations included mortality, moribundity, general health, and signs of toxicity. Clinical observations included skin and fur characteristics, eye and mucous membranes, respiratory, circulatory, autonomic, and central nervous systems, somatomotor, and behavior patterns. The body weight of each monkey was recorded before the start of the dosing period and at each sample collection time point with sedation.

Male and female K18-hACE2 Tg (strain #034860, B6.Cg-Tg[K18-ACE2]2Prlmn/J) mice were procured from The Jackson Laboratory (Bar Harbor, ME) and were ~8–10 weeks old at the time of vaccination. The vaccine dose in mice was 1.5 × 10^4^ PFU and the control group was immunized with 10 μg of spike-alum.

The animal studies were conducted in compliance with all relevant local, state, and federal regulations and were approved by the BIOQUAL Institutional Animal Care and Use Committee (IACUC).

RSV Memphis37b was kindly provided by hVIVO (United Kingdom) and RSV TN12/11-19, by R.S. Peebles Jr (Vanderbilt University Medical Center).

### Plasmid construction

The recombinant MV-014-212 and derived viruses were cloned in the antigenome orientation in bacterial artificial chromosomes (BAC) under the control of the T7 polymerase promoter^27^. The BACs containing the recombinant MV-014-212 and MVK-014-212 sequences were constructed by restriction digestion and ligation from the DB1-QUAD and kRSV-DB1-QUAD plasmids (encoding the antigenome of an attenuated version of RSV with or without the *mKate* gene, respectively)^60^. The DNA sequence encoding the chimeric spike protein, synthesized by Twist Bioscience was designed to contain compatible cloning sites. The kRSV-DB1-QUAD plasmid and spike insert were digested with the enzymes AatII and SalI (NEB) and ligated with T4 DNA ligase (NEB) overnight at 16 °C. Stabl3 chemically competent cells (Invitrogen) were transformed with the ligation mix and selected for chloramphenicol resistance for 20–24 h at 32 °C. MV-014-212 BAC was derived from the MVK-014-212 vector by removing the fragment between the KpnI and AatII restriction sites (~7 kb containing the *mKate* gene) and replacing it with the corresponding fragment extracted from DB1-QUAD by restriction digestion and ligation. For all the constructs, the sequences of the entire encoded viruses were confirmed by Sanger sequencing. Plasmid rA2-mkate was generated as follows: The pSynkRSV-line19F bacterial artificial chromosome (BAC) of A2-line19F RSV with the far-red fluorescent protein monomeric Katushka-2 (mKate2) in the first position was modified by replacing the line 19 strain fusion (F) gene, flanked by Sac II-to-Sal I sites in the BAC, with a synthetic cDNA (GeneArt) containing the A2 strain F open reading frame (A2 from Barney Graham, Vanderbilt University; GenBank accession number FJ614814) flanked by noncoding regions identical to those in pSynkRSV-line19F and corresponding Sac II-to-Sal I sites.

### Virus rescue and harvest

Vero cells were electroporated with the BAC encoding MV-014-212 (or the reporter virus) together with helper plasmids based on the pCDNA3.1 vector encoding the RSV proteins N, P, M2-1, and L, and the T7 polymerase under the control of a CMV promoter^27^. The cells were recovered in SFM-OptiPro medium supplemented with 4 mM glutamine and 10% FBS (Hyclone) for two passages and then expanded in serum-free medium with glutamine until CPE was extensive.

The recombinant viruses were harvested in Williams E medium (Hyclone) supplemented with sucrose phosphate glutamate (SPG) buffer or SPG buffer alone by scraping the infected cells directly into the media. The lysate was vortexed for 15 s at maximum speed (3200 rpm) to release the viral particles and then flash frozen. One cycle of thawing and vortexing was performed to increase the release of virus before the stocks were aliquoted, flash-frozen, and stored at –70 °C until use.

### Microscopy

The micrographs were captured with a DM IL LED Leica inverted fluorescence microscope and a Leica K5 camera using the acquisition software Leica application suite X, 3.7.2.22383.

### Immunofluorescence assay for antigenic characterization of MV-014-212

Vero cells were grown on borosilicate coverslips (VWR) in serum-free medium OptiPro (Gibco) supplemented with 4 mM of L-glutamine (Gibco) and antimycotic/antibiotic mix at 37 °C, 5% CO_2_, with 95% humidity. Subconfluent cells were infected with MV-014-212 in the same medium at an MOI of 0.01. Infection was allowed to proceed for 3 days at 32 °C and the cells were washed twice in PBS (Corning) and fixed in 4% paraformaldehyde in PBS (Alfa Aesar) for 15 min at room temperature. The cells were rinsed twice with PBS for 5 min and permeabilized with 0.1% Triton X-100 (VWR) in PBS for 10 min at room temperature. The cells were rinsed twice with PBS for 5 min and incubated in 10% normal goat serum in PBS (Invitrogen by Life Technologies) for 30 min at room temperature. The serum was discarded, and the infected cells were incubated at 37 °C with the monoclonal neutralizing antibodies anti-SARS-CoV-2 spike R0004 and MM43 (Sino Biological Inc., Beijing, China). at a concentration of 30 μg/mL in PBS. The cells were rinsed three times with PBS for 5 min and incubated with Alexa488-conjugated secondary antibodies (goat anti-rabbit and goat anti-mouse, respectively, both from Invitrogen) for 1 h at 37 °C in the dark. The secondary antibody was removed, and the cells were then incubated with 1 μg/mL DAPI (Invitrogen) for 5 min. Finally, the cells were washed twice in PBS and the coverslips were mounted onto glass slides with a drop of 9 parts glycerol (VWR) 1 part PBS. Images were acquired at a total magnification of ×200 with a Leica K5 camera and a DM IL LED Leica inverted fluorescence microscope.

### Plaque assay

Plaque assays for all the viruses used were performed in 24-well plates on Vero cells. Cells at 70% confluence were inoculated with 100 µL of 10-fold serial dilutions of viral samples (10^−1^ to 10^−6^). Inoculation was performed at room temperature with gentle rocking for 1 h before adding 0.75% methylcellulose (Sigma) dissolved in MEM supplemented with 10% FBS (Corning) and 1× Corning Antibiotic/Antimycotic mix. Cells were incubated for 4–5 days at 32 °C before fixing in methanol and immunostaining. For MV-014-212 and MVK-014-212, rabbit anti-SARS-CoV-2 spike polyclonal antibody (Sino Biological) and goat anti-rabbit horseradish peroxidase (HRP)-conjugated secondary antibody (Jackson ImmunoResearch) were used for immunostaining the plaques. For immunostaining rA2-mKate2, goat anti-RSV primary antibody (Millipore) and donkey anti-goat HRP-conjugated secondary antibody (Jackson ImmunoResearch) were used All viral plaques were developed for counting using 3-amino-9-ethylcarbazole (AEC) (Sigma). The limit of detection is 1 PFU per well, corresponding to a minimum detectable titer of 100 PFU/mL.

For detecting virus shedding in AGMs, the NS and BAL samples were collected and stored on ice until assayed for vaccine shedding by plaque assay without prior freezing.

### RNA sequencing

RNA from MV-014-212 samples was extracted using QIAamp^®^ Viral RNA Mini Kit following the manufacturer instructions (Qiagen). The quality and concentration of the extracted RNA were evaluated by gel electrophoresis and UV spectrophotometry. The extracted RNA was used as the template for reverse transcription using Invitrogen SuperScript^®^ IV First-Strand Synthesis System using a specific primer or random hexamers. The cDNA 2nd strand was synthesized with the Platinum^TM^ SuperFi^TM^ PCR Master Mix. The purified PCR products were directly sequenced using the BigDye^®^ Terminator v3.1 Cycle Sequencing Kit (Applied Biosystems). The sequencing reactions were purified using Sephadex G-50 purification and analyzed on ABI 3730xl DNA Analyzer. The sequence traces were assembled using Sequencher software and the consensus sequences were manually aligned with the reference sequence. The RNA sequencing for this study was performed by Avance Biosciences Inc., Houston, TX.

### Western blot

Viruses and control recombinant SARS-CoV-2 spike protein (LakePharma) were denatured with Laemmli sample buffer (Alfa Aesar) by heating at 95 °C for 10 min. Proteins were separated by SDS-PAGE in a 4–15% gradient gel and transferred to polyvinylidene fluoride membranes using a transfer apparatus according to the manufacturer’s protocol (BIO-RAD). After transfer, blots were washed in deionized water and probed using the iBind Flex system according to the manufacturer’s protocol (Invitrogen, Thermo-Fisher). Rabbit anti-SARS-CoV-2 spike (Sino Biological Inc., Beijing, China) was diluted in iBind solution (Invitrogen) at 1:1000. HRP-conjugated anti-rabbit IgG (Jackson ImmunoResearch) was diluted in iBind Solution at 1:5000. Blots were washed in deionized water and developed with Azure Biosystems Radiance Plus Femtogram HRP substrate (Azure Biosystems) according to manufacturer’s protocol. The blots were stripped with Restore Western Blot Stripping Buffer (Thermo-Fisher) and reprobed with goat anti-RSV polyclonal antisera (Sigma-Aldrich) and a monoclonal antibody specific for GAPDH (6C5) protein (Thermo-Fisher).

### RT-qPCR of SARS-CoV-2 subgenomic RNA for detecting shedding of challenge virus

Total RNA from tissues was extracted using RNA-STAT 60 (Tel-test “B”)/chloroform followed by precipitation of the RNA and resuspension in RNase-free water. To detect SARS-CoV-2 sgRNA, a primer set and probe were designed to detect a region of the leader sequence and *E* gene RNA from SARS-CoV-2. A standard curve was prepared using known quantities of plasmid DNA containing the *E* gene sequence, including the unique leader sequence, to produce a concentration range from 1 to 10^6^ copies/reaction. The PCR reactions were assembled using 45 μL master mix (Bioline) containing 2x buffer, Taq-polymerase, reverse transcriptase, and RNase inhibitor. The primer pair was added at 2 μM, and 5 μL of the sample RNA was added to each reaction in a 96-well plate. The PCR reactions were amplified in an Applied Biosystems 7500 Sequence detector using the following conditions: 48 °C for 30 min, 95 °C for 10 min followed by 40 cycles of 95 °C for 15 s, and 55 °C for 1 min. The limit of detection of the qPCR assay was 50 copies per mL.

Primers/Probe sequences are shown below:

SG-F: CGATCTTGTAGATCTGTTCCTCAAACGAAC

SG-R: ATATTGCAGCAGTACGCACACACA

FAM-ACACTAGCCATCCTTACTGCGCTTCG-BHQ.

### TCID50 assay to detect shedding of SARS-CoV-2 in BAL and NS of AGMs after challenge

Vero TMPRSS2 cells (obtained from Adrian Creanga, Vaccine Research Center-NIAID) were plated at 25,000 cells/well in DMEM + 10% FBS + gentamicin and the cultures were incubated at 37 °C and 5.0% CO_2_. Cells were 80–100% confluent the following day. Medium was aspirated and replaced with 180 μL of DMEM + 2% FBS + gentamicin. Twenty microliters of sample were added to the top row of a 96-well plate in quadruplicate and mixed using a P200 pipettor five times. Using pipettor, 20 μL representing 10-fold serial dilutions was transferred to the next row, and repeated down the plate (columns A–H). Positive (virus stock of known infectious titer) and negative (medium only) control wells were included in each assay set-up. The plates were incubated at 37 °C and 5.0% CO_2_ for 4 days. The cell monolayers were visually inspected for CPE. The TCID_50_ value was calculated using the Reed-Muench formula.

### SARS-CoV-2 total IgG ELISA for AGM sera

Prior to study initiation, AGMs were screened for spike-specific and nucleoprotein-specific IgG antibodies using commercial ELISA kits (Abcam). The assays were performed according to the manufacturer’s protocols. MaxiSorp immuno plates (Thermo-Fisher) were incubated overnight at 4 °C with 100 μL of 0.65 μg/mL of pre SARS-CoV-2 spike in PBS (Nexelis). The protein solution was removed, and the plate was washed four times with 250 μL of PBS supplemented with 0.05% Tween 20 (PBST). Blocking solution (PBST containing 5% milk) was added at 200 μL per well and the plate was incubated for 1 h at room temperature. A pool of human COVID-19 convalescent serum (Std/012020, Nexelis) was diluted in blocking solution and used as a standard. Serum samples and negative control serum were diluted at 1:25 followed by eight 2-fold serial dilutions in blocking solution. The blocking solution was removed from the plate and the wells washed once with 250 μL of PBST followed by addition of 100 μL of the diluted serum samples and controls. The plate was incubated for 1 h at room temperature. The plate was washed four times with 250 μL PBST and 100 μL of HRP-conjugated goat anti-monkey IgG antibody (Thermo-Fisher) diluted in blocking solution was added to each well following the last wash step. The plate was incubated for 1 h at room temperature and then washed four times in 250 μL PBST. Developing solution containing 3,3′,5,5′-Tetramethylbenzidine (TMB) substrate (1-Step Ultra TMB-ELISA Substrate Solution, Thermo-Fisher) was added to each well and the plate was incubated at room temperature for 30 min to allow the color to develop. The colorimetric reaction was terminated by the addition of 100 μL of ELISA Stop Solution (Invitrogen). The absorbance at 450 and 650 nm was read by spectrophotometry using a SpectraMax iD3 microplate reader (Molecular Devices).

### SARS-CoV-2 IgA ELISA for AGM nasal swabs

Purified prefusion SARS-CoV-2 spike antigen (LakePharma) was adsorbed onto 96-well MaxiSorp immuno microplate (Thermo-Fisher). The positive control was a serum pool from three COVID-19 convalescent individuals (Nexelis). Total IgA purified from human serum was used as a standard (Sigma-Aldrich). To generate the IgA standard curve anti-human IgA capture antibodies, Mab MT57 (Mabtech), were absorbed on plates instead of spike antigen. Following incubation, the microplate was washed four times with 250 µL PBST and blocked with 1% BSA in PBST. Purified human IgA standard, controls, or sample dilutions were then added and incubated in the coated microplate to allow binding. The plates were washed and a biotinylated goat anti-human IgA antibody (Mabtech) with cross-reactivity to monkey antibodies was added to all wells. Excess biotinylated anti-IgA antibody was removed by washing and streptavidin-conjugated HRP (Southern Biotech) was added. TMB was added and color development was stopped by addition of stop solution from Invitrogen. The absorbance of each well was measured at 450 nm. The standard total IgA antibody assayed on each test plate was used to calculate the concentration of IgA antibodies against spike protein in the AGM samples expressed in the arbitrary units ELU/mL. The measurements were performed in duplicate and average values are reported with standard deviations.

### Binding to hACE2

To study binding of MV-014-212 to hACE2, a whole virus ELISA was carried out using the surrogate virus neutralization assay kit from Genscript with a modified protocol. Virus samples at 2 × 10^5^ PFU/mL were diluted 1:2 in sample dilution buffer (SDB, Genscript) and incubated on the hACE2-coated capture plate for 30 min at 37 °C. The wells were washed 4 times with wash solution (Genscript) and incubated with rabbit anti-SARS-CoV-2 spike polyclonal antibody (Sino Biological) in blocking solution (5% nonfat milk in PBST) for 1 h at room temperature. The wells were washed 4 times with PBST and incubated with goat anti-rabbit horseradish peroxidase (HRP)-conjugated secondary antibody (Jackson ImmunoResearch) in blocking solution for 1 h at room temperature. The wells were then washed 4 times with PBST and incubated with TMB solution (Genscript) for 15 min in the dark at room temperature. The reaction was terminated with stop solution (Genscript) and absorbance at 450 nm was immediately read in a SpectraMax iD3 microplate reader (Molecular Devices). Spike protein (SARS-CoV-2) (LakePharma) was used as a positive control in a 1:10 serial dilution from 15.3 to 0.0153 μg/mL. Positive control samples were further diluted 1:10 in SDB. MV-012-968 (attenuated RSV strain) at 2 × 10^5^ PFU/mL was used as a negative control and diluted 1:2 in SDB.

### Pseudovirus neutralization assay (PNA)

SARS-CoV-2 spike ΔCT protein-bearing VSV pseudotyped particles in which the VSV glycoprotein *G* gene was replaced by the luciferase reporter gene were purchased from Nexelis, and the assay was performed at Meissa. Starting with a dilution of 1/25, serial 2-fold dilutions of heat-inactivated AGM sera were incubated with the pseudotyped viral particles (3 × 10^5^ RLU/well) at 37 °C for 1 h, after which the mixes were used to infect Vero E6 monolayers in white 96-well plates. The cells were incubated with the infection mixes for 20 ± 2 h at 37 °C in a 5% CO_2_ incubator. The medium was then discarded, and the cells were lysed in ONE-GloTM EX Luciferase Reagent (Promega). The lysis and luciferase reactions were allowed to proceed for 3 min at room temperature with shaking at 600 rpm and luminescence was read with a SpectraMax i3D plate reader. The values of luminescence were converted to percentage of inhibition using the equation:

1 $${{{\mathrm{\% }}}}\;{{{\mathrm{inhibition}}}} = 100 \ast \left[ {1 - \frac{{{{{\mathrm{L}}}} - {{{\mathrm{MIN}}}}}}{{{{{\mathrm{MAX}}}} - {{{\mathrm{MIN}}}}}}} \right]$$

where MIN is the average of reads obtained in the cell-only wells and MAX is the average of the reads from the wells of the pseudovirus-only control. L is the sample luminescence value. The inhibition vs. dilution curves were fitted using nonlinear regression, option “[inhibitor] vs. normalized response-variable slope” in GraphPad Prism (version 9.0.0). From the fitting IC_50_ was obtained and NT_50_ was calculated as the reciprocal of IC_50_. The convalescent serum used as a control for this assay was sourced from Nexelis. It constitutes a pool of three human convalescent sera collected from 21 May 2020 to 05 Jun 2020, with a titer of 1859 IU/mL.

### Plaque reduction neutralization test

A conventional 50% plaque-reduction neutralization test (PRNT_50_) was performed to measure neutralizing antibody titer as previously reported^41^. Briefly, individual sera were 2-fold serially diluted in culture medium with a starting dilution of 1:20 (dilution range of 1:20 to 1:320 for the samples, 1:80 to 1:1280 for the convalescent control). The diluted sera were incubated with 100 PFU of USA-WA1/2020 or mutant SARS-CoV-2. After 1 h of incubation at 37 °C, the serum-virus mixtures were used to infect monolayers of Vero E6 cells pre-seeded on 6-well plates on the previous day. A minimal serum dilution that suppresses >50% of viral plaques is defined as PRNT_50_.

All recombinant SARS-CoV-2s with spike mutations^42^ were prepared on the genetic background of an infectious cDNA clone derived from clinical strain USA-WA1/2020^61^.

The convalescent control in this assay is the same as described for the pseudovirus neutralization assay.

### Surrogate virus neutralization assay (sVNA)

The sVNA kit was purchased from Genscript and used according to the manufacturer’s recommendations. Briefly, the samples were diluted 1:10 in Sample Dilution Solution and incubated with HRP-conjugated spike RBD for 30 min at 37 °C to allow for the neutralization to occur. 100 μL of the neutralization mixes were then incubated for 15 min at 37 °C in a 96-well plate coated with human ACE2 protein. The wells were washed four times with 260 μL of Wash Solution and 100 μL of TMB were added to each well. The reaction was allowed to proceed at room temperature (20 °C ± 2 °C) in the dark and was stopped by the addition of 50 μL of Stop Solution per well. Absorbance at 450 nm was read immediately in a Spectramax id3 plate reader. A standard consisting of a proprietary mix of monoclonal antibodies (Genscript) was used to interpolate the neutralizing titers of the samples, as recommended by the manufacturer. The lower limit of quantification was calculated in each experiment as the value corresponding to an inhibition of 30%.

### Spike-specific IgG1 and IgG2a ELISA

Serum samples from mice were collected on day −21 and on day 28 post-vaccination to quantify the levels of SARS-CoV-2 spike-specific IgG1 and IgG2a antibodies by ELISA. Purified prefusion-stabilized SARS-CoV-2 spike protein (SARS-CoV-2/human/USA/WA1/2020, LakePharma) was diluted to 1 µg/mL in PBS and 100 μL was added to each well of a MaxiSorp immuno plate (Thermo-Fisher) and incubated overnight at 4 °C. The plate was washed four times in PBST and 100 μL of blocking solution (PBST + 2% BSA) was added to each well, and the plate was incubated for 1 h at room temperature. Serum dilutions were prepared in blocking solution with the first dilution at 1:25 for the IgG1 assay or 1:10–1:100 for the IgG2a assay. SARS-CoV-2 spike IgG1 (Sino Biological) or anti-spike-RBD-mIgG2a (InvivoGen) were diluted in blocking solution and used as standards for the assay.

The blocking solution was removed and 100 μL of diluted antibody added to each well. The plate was incubated at room temperature for 1 h and then washed four times in PBST using the plate washer. 100 μL of HRP-conjugated goat anti-mouse IgG1 (Thermo-Fisher) or HRP-conjugated goat anti-mouse IgG2a (Thermo-Fisher) secondary antibodies diluted 1:32,000 and 1:1000, respectively, were added to each well and the plate was incubated at room temperature for 1 h. The plate was washed four times in PBST. 100 μL of 1-step ultra TMB-ELISA substrate solution (Thermo-Fisher) was added to each well and the plate incubated for 30 min with constant rocking on an orbital shaker. After the incubation period, 100 μL of stop solution (Invitrogen) was added to each well and the plate was read using a Spectramax id3 plate reader (Molecular Devices) at 450 and 620 nm.

### ELISPOT of splenocytes from MV-014-212-vaccinated hACE2-mice

Spleens from vaccinated ACE2 mice were collected on day 28 post inoculation and stored in DMEM containing 10% FBS on ice until processed. The spleens were homogenized in a sterile petri dish containing medium. The homogenate was filtered through a 100-μm cell strainer and the cell suspension transferred to a sterile tube on ice. The cells were collected by centrifugation at 200 × *g* for 8 min at 4 °C. The supernatant was removed and residual liquid on the edge of the tube blotted with a clean paper towel. Red blood cells were lysed by resuspending the cell pellet in 2 mL of ammonium-chloride-potassium lysis buffer (155 mM ammonium chloride, 10 mM potassium bicarbonate, 0.1 mM ethylenediaminetetraacetic acid [EDTA]) and incubating the samples at room temperature for ~5 min. PBS was added at 2× to 3× the volume of cell suspension and cells were collected by centrifugation at 200 × *g* for 8 min at 4 °C. The cell pellet was washed twice in PBS and the cells were collected by centrifugation at 200 × *g* for 8 min at 4 °C. The supernatant was removed, and the pellet resuspended in 2 mM L-glutamine CTL-Test Media (Cell Technology Limited). The suspension was filtered through a 100-μm cell strainer into a new 15-mL conical tube and the cells were counted using a hemocytometer and resuspended at the appropriate cell concentration. Cells were maintained at 37 °C in a humidified incubator with 5% CO_2_ until used in the ELISpot assay.

The ELISpot assay was performed using a mouse IFNγ/IL-5 Double-Color ELISPOT assay kit (Cell Technology Limited). Murine IFNγ/IL-5 capture solution and 70% ethanol was prepared according to the manufacturer’s protocol. The membrane on the plate was activated by addition of 15 μL of 70% ethanol to each well. The plate was incubated for <1 min at room temperature followed by addition of 150 μL PBS. The underdrain was removed to drain the solution in the wells and each well was washed twice with PBS. Murine IFNγ/IL-5 capture solution (80 μL) was added to each well and the plate was sealed with parafilm and incubated at 4 °C overnight. The capture solution was removed, and the plate washed once with 150 μL PBS. A peptide pool containing peptides of 15 amino acids in length that span the SARS-CoV-2 spike protein (PepMix™ SARS-CoV-2 spike Glycoprotein, JPT Peptide Technologies) were prepared at 10 mg/mL and 100 μL was added to each well. A positive control containing concanavalin A (Con A) mitogen (10 μg/mL) was added to a separate reaction mixture. The splenocytes were mixed with CTL-Test™ Medium (Cell Technology Limited) to yield a final cell density of 3,000,000 cells/mL and 100 μL/well were added to the plate using large orifice tips. The plate was incubated at 37 °C in a humidified incubator containing 9% CO_2_ for 24 h. The plates were washed twice with PBS and then twice with PBST at a volume of 200 μL/well for each wash followed by addition of 80 μL/well anti-murine IFNγ/IL-5 detection solution. The plates were incubated at room temperature for 2 h. The plate was washed three times with PBST at 200 μL/well for each wash followed by the addition of 80 μL/well of tertiary solution. The plates were incubated at room temperature for 1 h. The plate was washed twice with PBST, and then twice with 200 μL/well of distilled water. Blue developer solution was added at 80 μL/well and the plate was incubated at room temperature for 15 min. The plate was rinsed three times in tap water to stop the developing reaction. After the final wash, red developer solution was added at 80 μL/well and the plate was incubated at room temperature for 5–10 min. The plate was rinsed three times to stop the developing reaction. The plate was air-dried for 24 h face-down on paper towels on the bench top. The spots on the plate representing splenocytes expressing IFNγ (red) or IL-5 (blue) were quantified using the CTL-immunospot plate reader (ImmunoSpot 7.0.23.2 Analyzer Professional DC\ImmunoSpot 7, Cellular Technology Limited) and software (CTL Switchboard 2.7.2).

### Statistics

All statistical analysis was performed in GraphPad Prism 9.0 Software for Windows (GraphPad Software). Datasets were tested for normality using Shapiro–Wilk test prior to selecting an appropriate parametric or non-parametric statistical test. Analysis of Variance (ANOVA) was performed with multiple comparison using Tukey’s correction when all groups were compared and Šidák’s correction when pre-selected pairwise analysis was used. Student’s *t*-test was performed for unpaired data with no additional correction. Area under the curve was calculated per animal for log10-transformed viral loads and baseline was set to assay limit of detection.

### Reporting summary

Further information on research design is available in the Nature Research Reporting Summary linked to this article.

## Supplementary information

Supplementary Figures 1 to 11

REPORTING SUMMARY

## Data Availability

The sequences of the antigenomes of MV-014-212 and MVK-014-212 are deposited in GenBank with accession numbers MZ695841 and MZ695842. The datasets generated in the present study are available from the corresponding author on reasonable request.
