# Supplementary material for: Mucosal administration of a live attenuated recombinant COVID-19 vaccine protects nonhuman primates from SARS-CoV-2
Source: NPJ Vaccines. 2022 Jul 29;7:85. doi: 10.1038/s41541-022-00509-6 (PMC9334537; doi:10.1038/s41541-022-00509-6)
Supplement: Supplementary file 1 — Supplementary Figures 1 to 11 [file 41541_2022_509_MOESM1_ESM.pdf]

Supplementary Information

Mucosal administration of a live attenuated recombinant COVID-19 vaccine protects nonhuman primates from SARS-CoV-2

Mariana F. Tioni, Robert Jordan, Angie Silva Pena, Aditya Garg, Danlu Wu, Shannon I. Phan, Christopher M. Weiss, Xing Cheng, Jack Greenhouse, Tatyana Orekov, Daniel Valentin, Swagata Kar, Laurent Pessaint, Hanne Andersen, Christopher C. Stobart, Melissa H. Bloodworth, R. Stokes Peebles Jr, Yang Liu, Xuping Xie, Pei-Yong Shi, Martin L. Moore and Roderick S. Tang

Supplementary Fig. 1 Other vaccine candidates designed in this study. The sequences of the C-termini of the candidates show the different positions of the junction between the respiratory syncytial virus (RSV) F protein cytoplasmic tail (red letters) and the SARS-CoV-2 spike protein transmembrane domain (blue letters). For reference, the full-length sequences of the C-termini of spike and F are shown above and below the candidate sequences, respectively. The candidates were designed to contain an *mKate2* gene as a fluorescent marker to facilitate monitoring of virus rescue and propagation in tissue culture. Candidates were evaluated for their ability to be rescued (defined as the generation of red fluorescent foci) and to grow to titers of 10^5 pfu/mL or higher. MV-014-212 was selected for further investigation. TM, transmembrane.

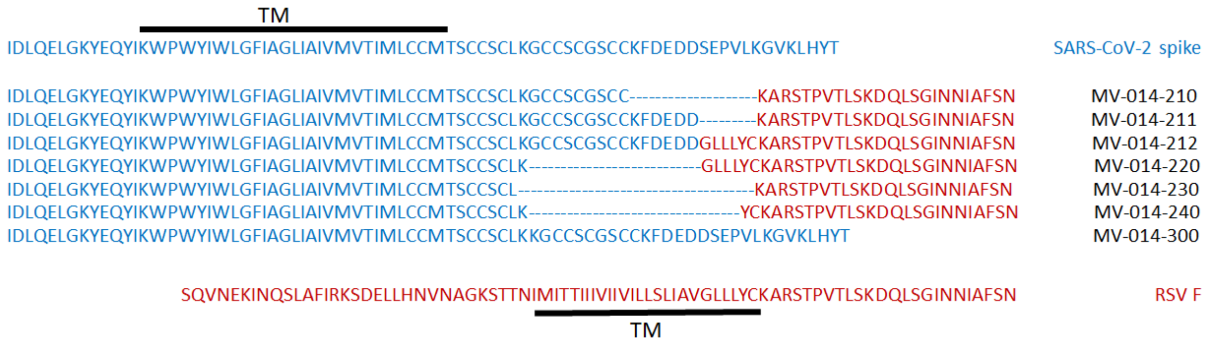

Vaccine candidate	Rescue	Achieved titers ≥ 10^5 PFU/mL
MV-014-210	Yes	Yes
MV-014-211	Yes	Not Determined
MV-014-212	Yes	Yes
MV-014-220	Yes	Not Determined
MV-014-230	No	No
MV-014-240	No	No
MV-014-300	Yes	No

Supplementary Fig. 2 Schematic of spike and its glycosylation sites and detection of SARS-CoV-2 spike expression by Western blotting. Based on its amino acid sequence, the calculated molecular weight of the chimeric spike protein monomer in MV-014-212 prior to glycosylation is approximately 143 kDa, (https://www.bioinformatics.org/sms/prot_mw.html). Wild-type non-glycosylated spike has a calculated molecular weight of 141.2 kDa, whereas the apparent molecular weight is approximately 180 kDa[1]. The chimeric protein encoded by MV-014-212 is expected to be glycosylated and showed the expected apparent size in a sodium dodecyl-sulfate polyacrylamide gel electrophoresis (SDS-PAGE). On proteolysis at the Subunit 1-Subunit 2 cleavage site, MV-014-212 spike would result in two subunits of 683 and 607 amino acids, respectively. Glycosylated Subunit 1 and Subunit 2[2] would be indistinguishable by SDS-PAGE. The figure below shows a schematic of spike and its glycosylation sites (vertical black bars) as reported by Watanabe et al[2]. NTD, N-terminal domain; RBD, receptor-binding domain; TM, transmembrane.

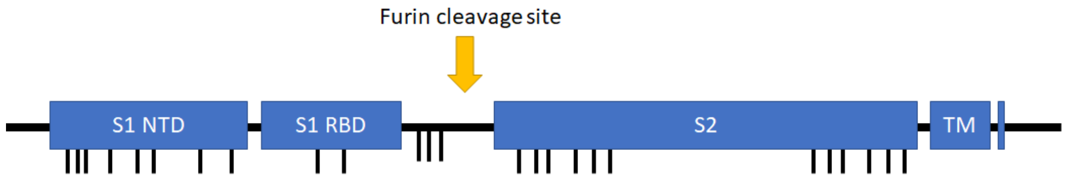

Uncropped Western blot showing full-length purified SARS-CoV-2 spike protein lacking the furin cleavage site (lane 1) MVK-014-212 (lane 2), MV-014-212 (lane 3), mock-infected Vero cell lysate (lane 4), blank (lane 5). Molecular weight markers showed the migration of the BIO-RAD Precision Plus Protein Dual Color Standards. The blue arrow indicated the expected size of the full-length spike and red arrow indicated the expected size of the cleaved protein (S1+S2). The blots were derived from the same experiment and processed in parallel. (A) probed with anti-SARS-CoV-2 polyclonal antibodies (B) probed with anti-RSV polyclonal

antibodies and (C) probed with anti-GAPDH monoclonal antibodies.

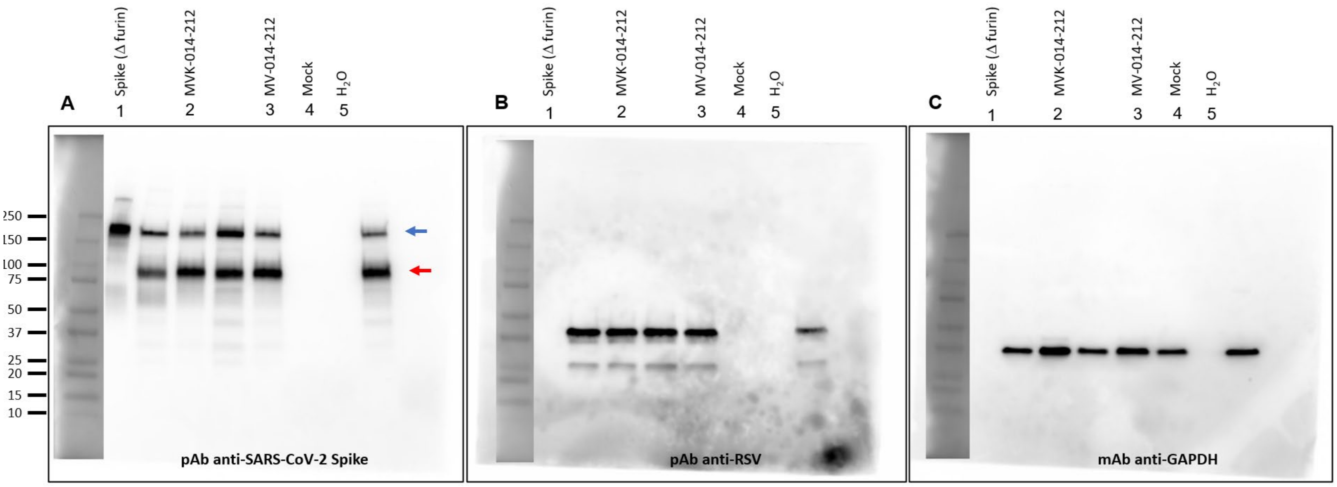

Supplementary Fig. 3. Antigenic characterization of MV-014-212 Spike

(a) Immunofluorescence staining of Vero cells infected with MV-014-212 using monoclonal neutralizing antibodies against SARS-CoV-2 spike. The top panels show cells treated with the rabbit monoclonal antibody R0004 (Sinobiological) and the bottom panels show cells treated with the mouse monoclonal antibody MM43 (Sinobiological). DAPI: 4′,6-diamidino-2-phenylindole. Scale bars are 100 µm. All images were acquired at a total magnification of 200x with a Leica K5 camera. Images were adjusted for brightness using Microsoft PowerPoint software. The adjustment was applied equally to the entire image in each panel.

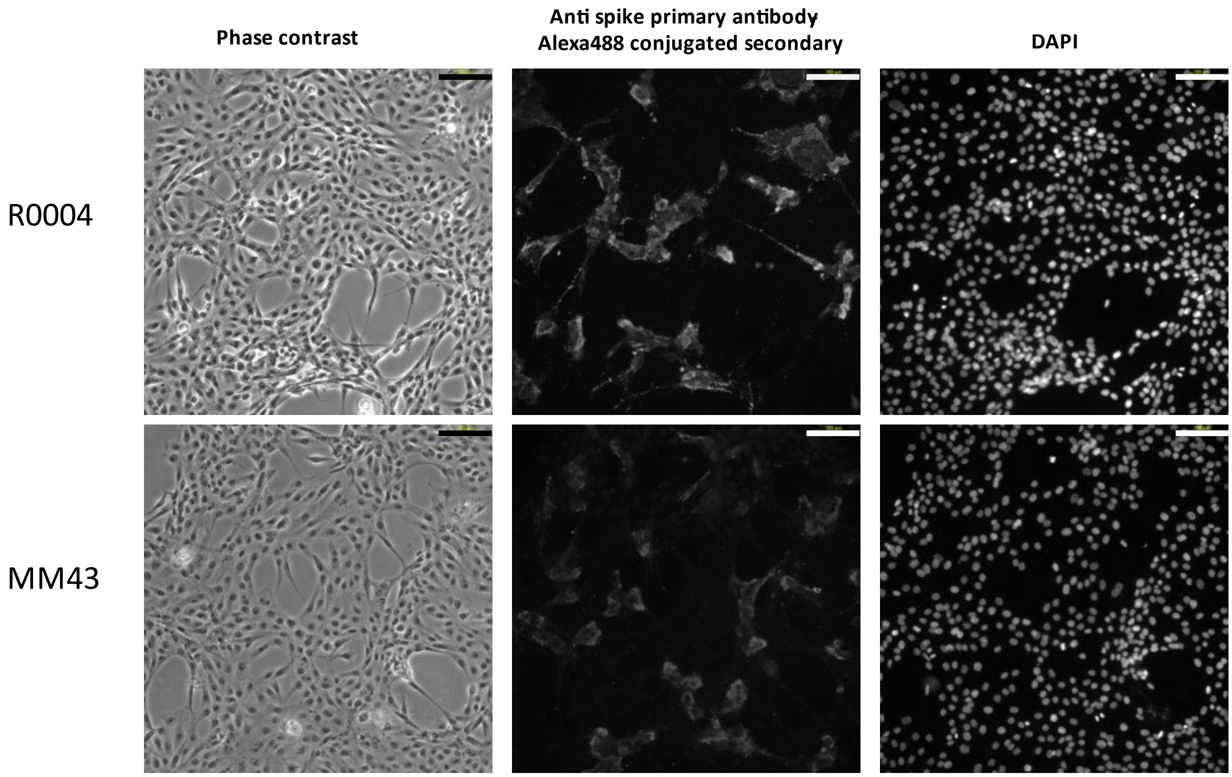

(b) Binding of MV-014-212 to human ACE2 (hACE2). Binding to (hACE2) was assessed using a whole-virus ELISA as described in methods. hACE2-coated plates were incubated with MV-014-212, and developed using a rabbit anti-spike polyclonal antibody, as primary, and an HRP-Goat anti rabbit IgG, as secondary antibody. Absorbance at 450 nm was read after reaction with a chromogenic substrate. The ELISA was performed using the hACE2-coated plates provided in the surrogate virus neutralization assay kit (Genscript). Control plates showed no non-specific binding of virus or detection antibodies to uncoated wells.

MV-012-968 was used as a specificity control in this assay. MV-012-968 is Meissa's RSV vaccine. It shares the general backbone of MV-014-212 but expresses RSV F and G proteins instead of Spike protein as its surface glycoproteins. S1 to S4 are serial dilutions of recombinant spike protein (LakePharma) from 15.3 μg/mL to 0.0153 μg/mL. They were used as a positive control in this assay. PBS: phosphate buffered saline served as a negative control. Bars represent the mean and error bars represent SD.

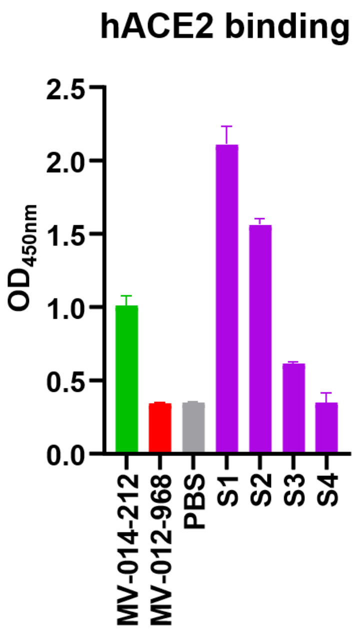

Supplementary Fig. 4 Spike and nucleoprotein-specific IgG antibody responses in pre-vaccinated African green monkeys.

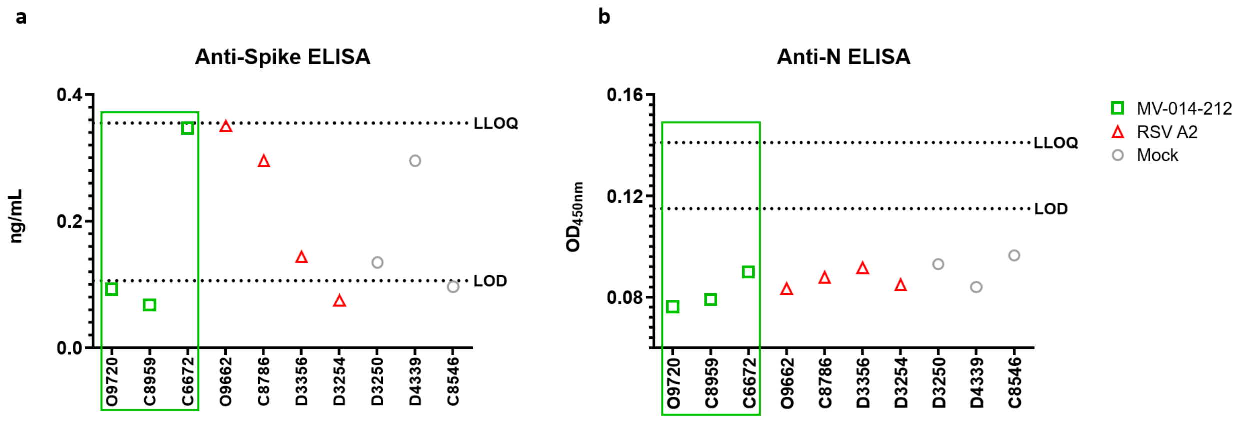

(a) Spike-specific and (b) nucleoprotein-specific serum IgG antibodies were measured by ELISA using serum collected from AGMs prior to study initiation. The anti-spike titer is expressed as ng/mL and the anti-N titer is expressed as OD units. Each symbol corresponds to one animal (N=3 AGMs for the MV-014-212 and mock groups and N=4 for the RSV A2 group). The study number of each AGM is shown. AGMs that will subsequently be administered MV-014-212 vaccine are in green boxes. LOD: limit of detection. LLOQ: lower limit of quantification.

Supplementary Fig. 5. AGM body weight in kg post-immunization and post-challenge.

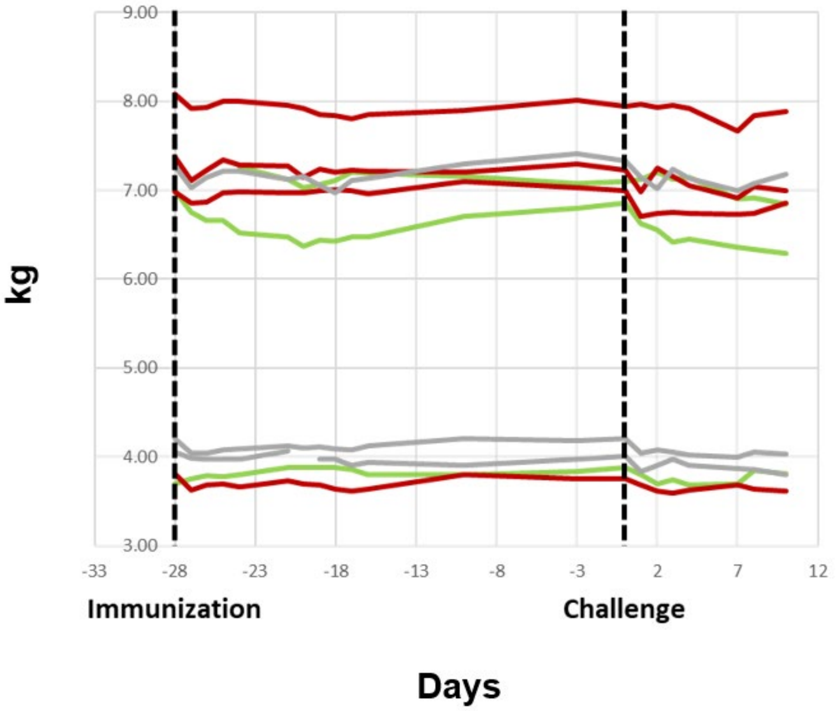

AGMs inoculated with MV-014-212 are shown in green, RSV A2 in red, and mock-infected in grey. Each line represents an individual animal.

Supplementary Fig. 6. AGM body temperature in Fahrenheit (°F) post-immunization and post-challenge.

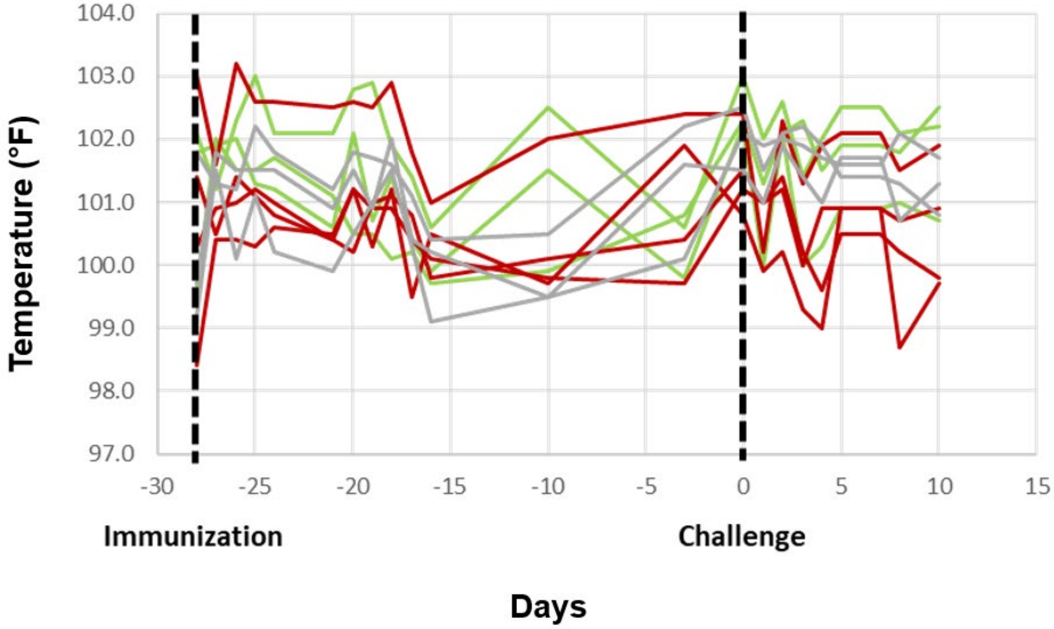

AGMs inoculated with MV-014-212 are shown in green, RSV A2 in red, and mock-infected in grey. Each line represents an individual animal.

Supplementary Fig. 7 Shedding kinetics of individual African green monkeys (AGMs) after vaccination. Panels (a and c) AGMs vaccinated with MV-014-212, panels (b and d) control group AGMs vaccinated with respiratory syncytial virus (RSV). (a) and (b) are nasal swab samples and (c) and (d) are bronchoalveolar lavage samples. The dotted lines indicate LOD. N=4 AGMs for the MV-014-212 and RSV A2 inoculated groups. The study number of individual AGMs are shown.

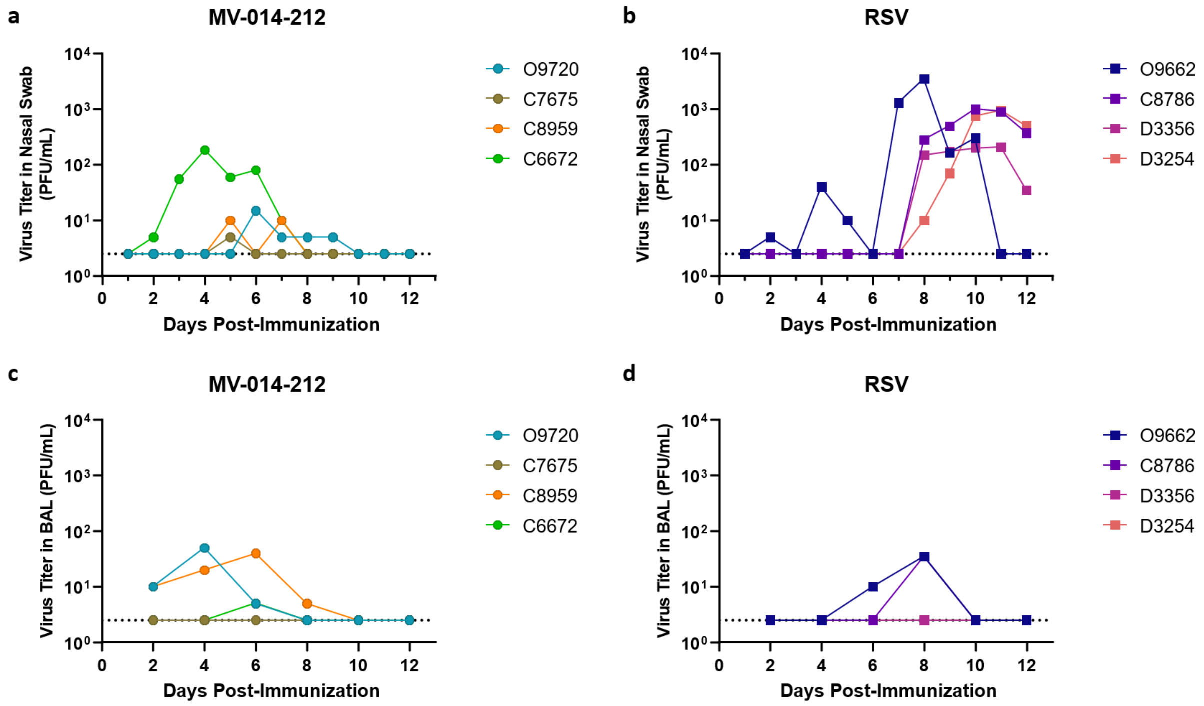

Supplementary Fig. 8 Two independent experiments were performed in cotton rats. In experiment 1, cotton rats (*n* = 5 per group) were inoculated with 1 × 10^5 pfu of biologically derived respiratory syncytial virus (RSV) TN 12/11-19 (TN12)[3], Memphis 37b (M37)[4], or recombinant A2 (rA2) RSV strains. On days 3, 5, and 7, cotton rat nasal and lung tissues were homogenized in Hank's balanced salt solution (HBSS) + 10% sucrose phosphate glutamate (SPG) buffer for titer determination by plaque assay. In this experiment only day 5 nasal and lung tissues were collected for the rA2 group. In experiment 2, cotton rats (*n* = 6) were inoculated with 5 × 10^5 pfu of rA2. On days 2, 5, and 7, cotton rat nasal and lung tissues were homogenized in HBSS +10% SPG buffer for titer determination by plaque assay. Plaque assay was performed in HEp-2 cells using clarified nasal and lung homogenates diluted in Eagle's minimum essential medium. Plaques were visualized by immunostaining with RSV polyclonal antibodies in experiment 1 and by crystal violet staining in experiment 2. Panel **a** shows replication kinetics of TN12, M37, and rA2 in the nose. Panel **b** compares nasal titers of TN12, M37, and rA2 on day 5 of experiment 1. Panel **c** shows replication kinetics of biological TN12 and M37 and rA2 in the lungs. Panel **d** compares lung titers on day 5 of experiment 1 for TN12, M37, and rA2. The result showed that the rA2 was attenuated in nose and lungs with the attenuation in the lung being more pronounced, at approximately 2 log difference relative to biologically derived RSV strains. For panels a and c, each data point represents the mean of five animals. For panels b and d, each data point represents one animal, and the horizontal bar represents the mean. Error bars indicate SD. *wt*, wild type. Statistical analysis is one-way ANOVA with Šídák's multiple comparisons test. *P*-values are indicated above the brackets.

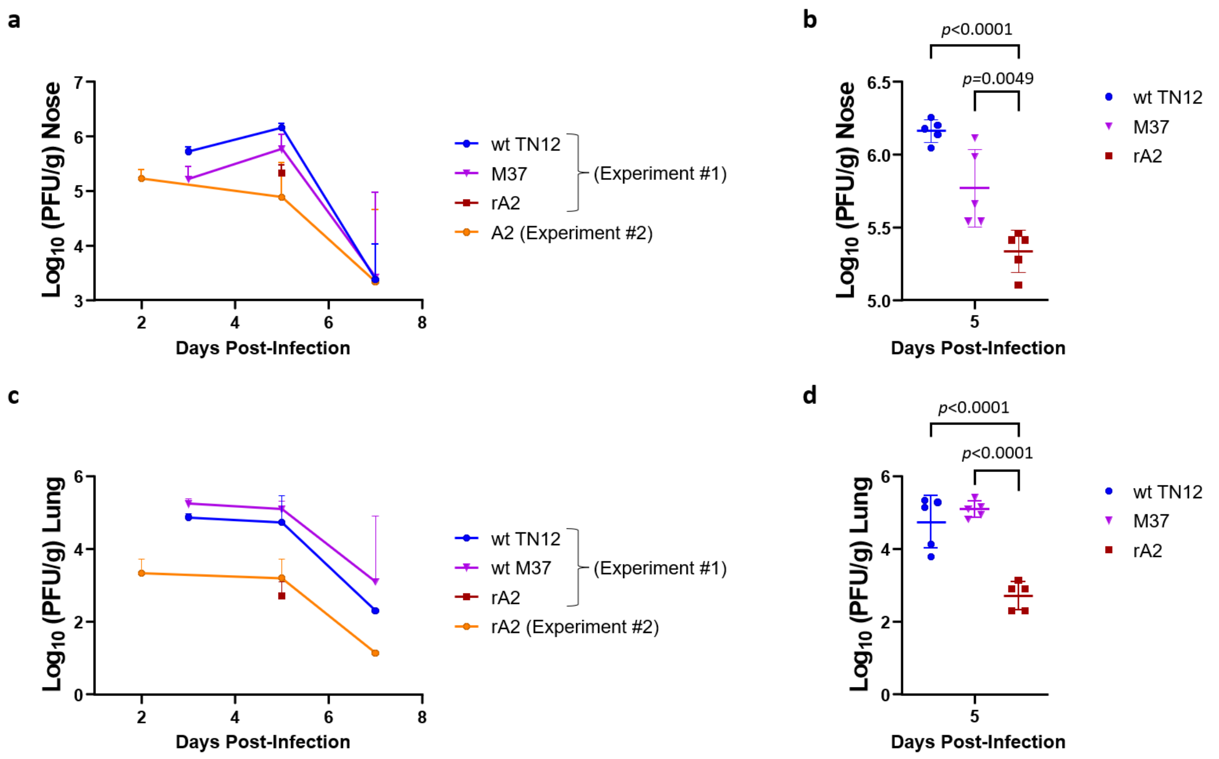
a
Log10 (PFU/g) Nose
7
6
5
4
3
2
4
6
8
Days Post-Infection
wt TN12
M37
rA2
A2 (Experiment #2)
(Experiment #1)
b
p<0.0001
p=0.0049
Log10 (PFU/g) Nose
6.5
6.0
5.5
5.0
5
Days Post-Infection
wt TN12
M37
rA2
c
Log10 (PFU/g) Lung
6
4
2
0
2
4
6
8
Days Post-Infection
wt TN12
wt M37
rA2
rA2 (Experiment #2)
(Experiment #1)
d
p<0.0001
p<0.0001
Log10 (PFU/g) Lung
6
4
2
0
5
Days Post-Infection
wt TN12
M37
rA2

Supplementary Fig. 9 SARS-CoV-2 shedding kinetics in individual African green monkeys after challenge by sgRNA. Panels (a and d) AGMs vaccinated with MV-014-212, panels (b and e) AGMs inoculated with respiratory syncytial virus (RSV), and panels (c and f) AGMs that were mock-inoculated. (a), (b) and (c) are nasal swab samples and (d), (e) and (f) are bronchoalveolar lavage samples. The dotted lines indicate LOD. N= 3 AGMs for the MV-014-212 and mock groups and N=4 AGMs for the RSV A2 group. The study number of individual AGMs are shown.

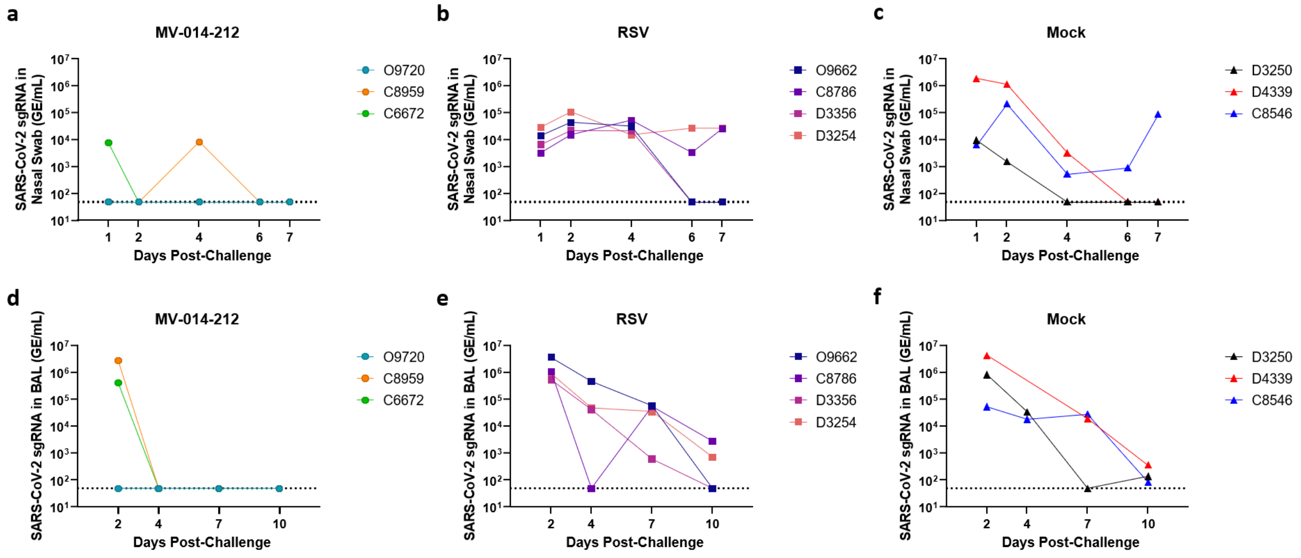

Supplementary Fig. 10 Median $TCID_{50}$ of SARS-CoV-2 shedding. (a) nasal swabs and (b) bronchoalveolar lavage (BAL) samples. Lines indicate mean and the error bars are standard deviation. (c) to (h) time course for individual animals in the study. N=3 AGMs for the MV-014-212 and mock groups and N=4 AGMs for the RSV A2 group. AGM, African green monkey; RSV, respiratory syncytial virus. $TCID_{50}$, median tissue culture infectious dose. LOD is indicated by dotted lines. Area under the curve for the shedding kinetics of each animal are shown in (i) for nasal swabs and (j) for BAL. Statistical analysis is one-way ANOVA, bars indicate mean, error bars are SD and the values above the brackets are p-values.

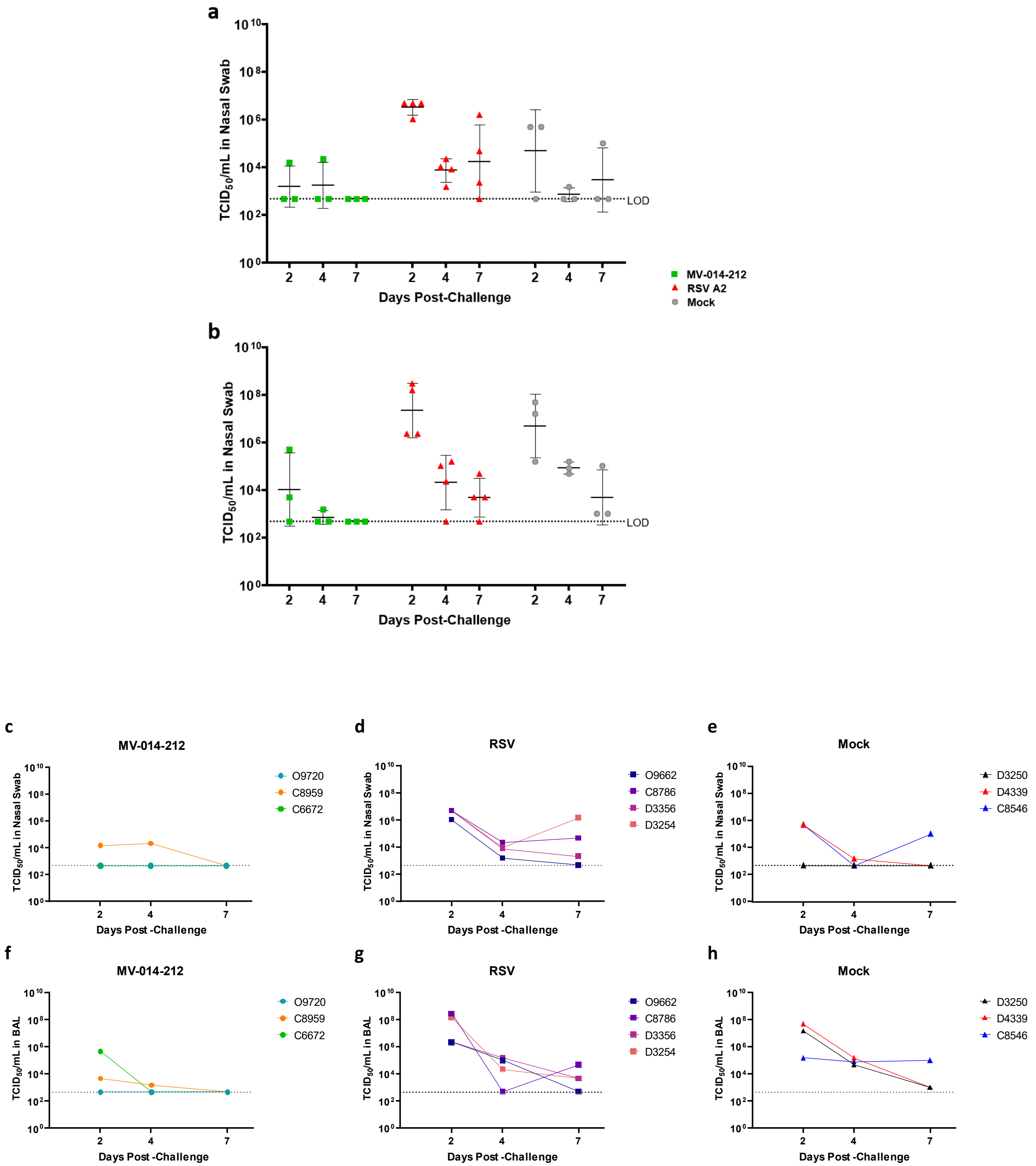
a
TCID50/mL in Nasal Swab
LOD
Days Post-Challenge
MV-014-212
RSV A2
Mock
b
TCID50/mL in Nasal Swab
LOD
Days Post-Challenge
c
MV-014-212
TCID50/mL in Nasal Swab
Days Post -Challenge
O9720
C8959
C6672
d
RSV
TCID50/mL in Nasal Swab
Days Post -Challenge
O9662
C8786
D3356
D3254
e
Mock
TCID50/mL in Nasal Swab
Days Post -Challenge
D3250
D4339
C8546
f
MV-014-212
TCID50/mL in BAL
Days Post -Challenge
O9720
C8959
C6672
g
RSV
TCID50/mL in BAL
Days Post -Challenge
O9662
C8786
D3356
D3254
h
Mock
TCID50/mL in BAL
Days Post -Challenge
D3250
D4339
C8546

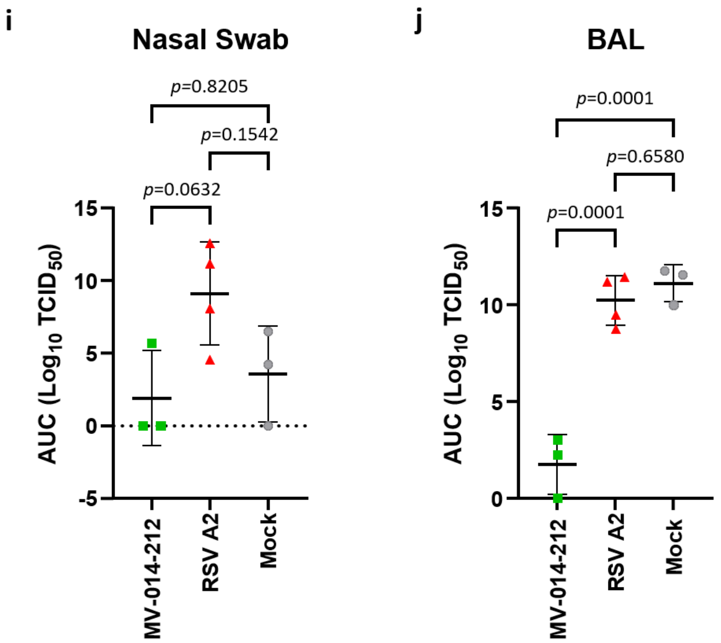

Supplementary Fig. 11 Absolute and normalized IgA in AGM nasal swabs at day 25 post vaccination. The values obtained in the spike-specific IgA ELISA were calculated as (a) ELISA units per mL (ELU/mL) and normalized by (b) total protein (determined using the Pierce™ BCA Protein Assay Kit, following the manufacturer's recommendation), or normalized by (c) urea (determined using the Sigma-Aldrich Urea Assay Kit). For urea normalization, the urea concentration in nasal swab samples and serum samples collected on the same day was measured. The ratio of the urea concentration in sera was divided by the urea concentration in the nasal swabs. This ratio was multiplied with the IgA concentration (ELU/mL) measured to obtain the urea normalized IgA concentration[5]. The different methods of analyzing the data resulted in the same trend across groups. Statistical analysis was one-way ANOVA using Tukey's multiple comparisons test. Values shown indicate *p*-values and the bar is geometric mean and error bars are geometric standard deviation. N= 3 AGMs for the MV-014-212 and mock groups and N=4 AGMs for the RSV A2 group. IgA responses for individual AGMs are indicated as (*1*) O9720, (*2*) C8959 and (*3*) C6672.

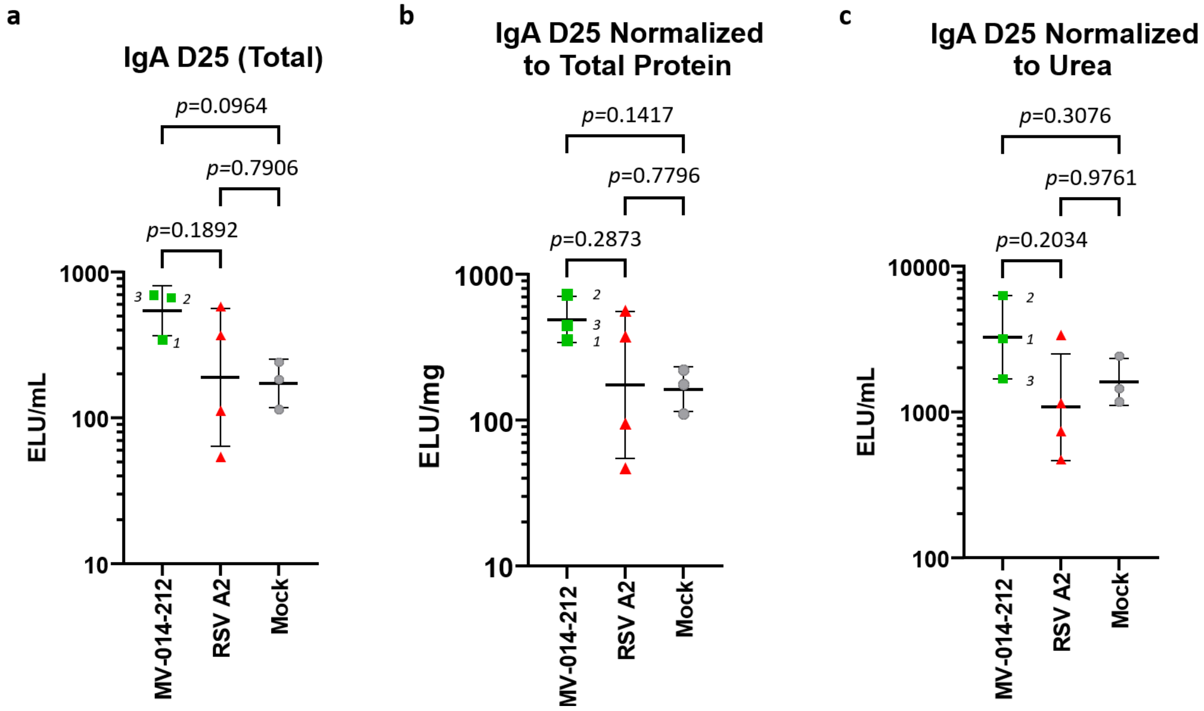
a
IgA D25 (Total)
p=0.0964
p=0.7906
p=0.1892
ELU/mL
1000
100
10
MV-014-212
RSV A2
Mock
b
IgA D25 Normalized to Total Protein
p=0.1417
p=0.7796
p=0.2873
ELU/mg
1000
100
10
MV-014-212
RSV A2
Mock
c
IgA D25 Normalized to Urea
p=0.3076
p=0.9761
p=0.2034
ELU/mL
10000
1000
100
MV-014-212
RSV A2
Mock

References

1. Ou, X. et al. Characterization of spike glycoprotein of SARS-CoV-2 on virus entry and its immune cross-reactivity with SARS-CoV. *Nat. Commun*.**11**, 1620 (2020).
2. Watanabe, Y., Allen, J.D., Wrapp, D., McLellan, J.S. & Crispin, M. Site-specific glycan analysis of the SARS-CoV-2 spike. *Science* **369**, 330-333 (2020).
3. Stier, M.T. et al. Respiratory syncytial virus infection activates IL-13-producing group 2 innate lymphoid cells through thymic stromal lymphopoietin. *J. Allergy Clin. Immunol*.**138**, 814-824.e11 (2016).
4. Kim, Y.I. Respiratory syncytial virus human experimental infection model: provenance, production, and sequence of low-passaged memphis-37 challenge virus. *PLoS One* **9**, e113100 (2014).
5. Habibi, M.S. et al. Impaired antibody-mediated protection and defective IgA B-cell memory in experimental infection of adults with respiratory syncytial virus. *Am. J. Respir. Crit. Care Med.* **191**, 1040-1049 (2015).
